# Mutual Information Regularization for Cross-Subject Gesture Recognition Using Surface Electromyography and Acceleration

**DOI:** 10.3390/s26206394

**Published:** 2026-10-09

**Authors:** Anyuan Zhang, Haowen Zheng, Yan Wu

**Affiliations:** School of Computer Science and Technology, Changchun University of Science and Technology, Changchun 130022, China; zhanganyuan@cust.edu.cn (A.Z.); 13331447552@163.com (H.Z.)

**Keywords:** surface electromyography, acceleration, gesture recognition, mutual information regularization, cross-modal representation learning, cross-subject transfer, multimodal fusion

## Abstract

Feature fusion enables joint classification of surface electromyography (sEMG) and acceleration (ACC) signals, but classification supervision alone does not explicitly optimize statistical dependence between their learned representations. To address this gap, we propose a dual-branch gesture recognition method that combines supervised classification with mutual information (MI) regularization. A Mutual Information Neural Estimation (MINE)-based dependence regularizer encourages cross-modal dependence during training; the auxiliary projection and critic are removed at inference. The experiments included 11 participants performing 10 gestures under four postures across five repetitions, with within-subject testing and strict leave-one-subject-out evaluation. The method achieved an accuracy/Macro-F1 of 93.78%/93.74% within subjects, 37.02%/34.72% before cross-subject adaptation, and 85.61%/85.57% after adaptation using one target repetition of 40 gesture–posture trials (9 min 20 s of scheduled recording). Compared with the Basic sEMG–ACC fusion baseline using the same recognition architecture without MI regularization, accuracy increased by 2.52, 6.00, and 3.06 percentage points in the three settings, respectively; all matched comparisons remained significant after Holm correction. After one-repetition adaptation, MI also outperformed the evaluated local implementations of MSCNN-TL and an ACC-adapted Multistream CNN with fine-tuning. MI achieved higher mean performance than cross-temporal attention alone (CTA-only) and its combination with MI (CTA+MI), although the cross-subject differences were not significant after Holm correction. These results support MI regularization as an effective addition to classification supervision in the evaluated cross-subject sEMG–ACC framework. The current evaluation focuses on offline steady-action classification and does not cover continuous interaction scenarios such as gesture detection and transition handling.

## 1. Introduction

Wearable gesture recognition has considerable potential for prosthetic control, rehabilitation assistance, and natural human–computer interaction. Surface electromyography (sEMG) provides a noninvasive means of recording electrical activity associated with muscle activation and has been widely used in upper-limb motion recognition, gesture classification, and myoelectric control [1,2,3]. Despite substantial advances in deep learning-based sEMG recognition, practical deployment remains challenging because models trained on one group of users often generalize poorly to unseen individuals. Differences in muscle anatomy, skin impedance, electrode placement, fatigue, motion artifacts, and movement execution can produce substantial inter-subject distribution shifts [4,5,6]. Collecting extensive labeled data for every new user is also time-consuming. Improving direct cross-subject recognition while limiting the target-user calibration burden therefore remains an important problem.

This study investigates cross-subject gesture decoding in healthy adults, with potential relevance to the decoding component of prosthetic and rehabilitation systems.

Convolutional models have been used for prosthetic hand-movement classification [7], supported by the NinaPro datasets [8,9]. Other studies have modeled instantaneous spatial sEMG patterns [10], combined convolutional, recurrent, and attention components [11] or used temporal convolutional designs [12]. Transfer learning addresses user-specific calibration and cross-subject variability [13]. Relevant transfer methods include the multiscale convolutional framework of Fratti et al. [14], the multistream sEMG–IMU fine-tuning framework of Li et al. [15], and domain adaptation and self-recalibration approaches [16,17,18,19,20]. These approaches address representation learning and inter-subject distribution mismatch.

Multimodal wearable sensing provides a way to combine physiological and kinematic information. Accelerometer signals, often acquired as part of an inertial measurement unit, describe limb motion and orientation [21,22,23,24], whereas sEMG records electrical activity associated with muscle activation. The modalities differ in sampling rate, physical origin, noise characteristics, and temporal dynamics. Separate encoders can accommodate these differences, and feature concatenation provides a direct route to joint classification.

Cross-attention has been applied to sEMG–accelerometer fusion for armband-based gesture recognition [25]. In cross-temporal attention (CTA), queries from one modality weight the values of the other according to query–key compatibility [26]. Although CTA enables adaptive cross-modal interactions, classification loss alone provides no explicit objective for optimizing statistical dependence between the modality encodings.

Mutual information (MI) offers a way to formulate such an objective. MI-based representation learning has used dependence maximization across views to learn useful features [27], while Mutual Information Neural Estimation (MINE) provides a trainable estimator for high-dimensional representations [28]. These studies motivate an explicit dependence-learning objective for the sEMG and ACC encoders, alongside supervised gesture classification. The roles are complementary: classification supervision targets gesture prediction, whereas MI regularization encourages dependence between the modality-specific representations. This training objective can be used without introducing an attention module into the fusion architecture.

In this work, MI regularization denotes the implemented MINE-based dependence regularizer. Shared dependence may also reflect posture, participant identity, or acquisition characteristics; encouraging it does not establish gesture-specific or subject-invariant representations.

We therefore propose a dual-branch sEMG–ACC recognition method that combines supervised classification with MINE-based MI regularization. The central hypothesis is that explicitly encouraging cross-modal dependence improves cross-subject gesture recognition relative to an otherwise identical model trained with classification loss alone, without requiring attention-based fusion. The original study also anticipated complementary gains from adding CTA to MI. The ablation results did not support this expectation: CTA+MI did not improve mean recognition performance over MI alone. The matched MI–Basic comparison assesses the added training objective, whereas CTA-only and CTA+MI examine attention-based fusion and its combination with MI regularization. The regularizer operates on pooled modality-specific encoder representations through an auxiliary projection and critic. These components are used only during training; inference retains separate pooling, feature concatenation, and classification. Multi-source pretraining and subsequent target-subject adaptation address transfer to an unseen user.

The experiments use a self-collected dataset with 11 participants, 10 gestures, four forearm postures, and five repetitions. Strict leave-one-subject-out evaluation excludes each target participant from source pretraining and measures recognition before and after adaptation. One complete target repetition, comprising 40 gesture–posture trials, is used for adaptation, and the remaining repetitions are reserved for testing. Within-subject evaluation provides a complementary setting. Transfer-learning baselines, an equal-budget source-diversity experiment, and auxiliary diagnostics provide further evaluation.

The main contributions are as follows:

A dual-branch sEMG–ACC recognition method supplements classification supervision with an explicit, MINE-based cross-modal dependence objective. Its auxiliary projection and critic are used only during training, preserving a concatenation-and-classification inference path.

A matched ablation against the same backbone without MI assesses the contribution of the regularizer. Comparisons with CTA-only and CTA+MI evaluate MI without attention and test the additional value of attention-based fusion.

Cross-subject evaluation before and after one-repetition adaptation, transfer-learning baselines, and within-subject tests characterize recognition performance. An equal-budget experiment assesses the tested source-subject compositions. Auxiliary analyses further characterize representation and prediction behavior across configurations.

Section 2 describes the proposed MI method and adaptation protocol before introducing the comparison configurations. Section 3 specifies the evaluation, implementation, sensitivity analysis, and statistical methods. Section 4 presents gesture-wise recognition, overall and posture-wise cross-subject performance, the matched MI ablation, attention extensions, source compositions, auxiliary diagnostics, and inference cost. Section 5 and Section 6 discuss the findings and conclude the paper.

## 2. Materials and Methods

### 2.1. Dataset and Data Acquisition

A synchronized multimodal sEMG–ACC dataset was collected for the within-subject and cross-subject evaluations conducted in this study. Eleven healthy, right-handed adults aged 20–27 years (mean ± SD: 23.4 ± 2.1 years) participated, including eight male and three female participants. None reported a history of neurological or musculoskeletal disorders. All participants were informed of the experimental procedure and provided informed consent before data acquisition. The experimental protocol was approved by the Institutional Ethics Committee of Changchun University of Science and Technology. The collected data were de-identified before analysis.

sEMG and ACC signals were synchronously recorded from the right forearm using a commercially available Sichiray wearable armband (Wuxi Sichiray Technology Co., Ltd., Wuxi, China). The device incorporated eight sEMG channels and a tri-axial accelerometer, enabling simultaneous acquisition of myoelectric activity and forearm-motion signals. The sEMG and ACC sampling rates were 500 Hz and 50 Hz, respectively.

The armband was positioned on the proximal forearm near the elbow, at a comfortable position for each participant, with consistent relative electrode positioning across participants. For each participant, it remained in place during acquisition and was not removed and re-fitted between repetitions. During preprocessing, temporal correspondence was established using the 500 Hz/50 Hz sampling-rate ratio and sample-index mapping; ACC samples and windows were aligned to the corresponding sEMG windows using the 10:1 ratio.

The experimental protocol comprised ten gestures: hand open, fist, wrist flexion, wrist extension, forearm pronation, forearm supination, thumbs-up, OK sign, index-finger extension, and V sign. Each gesture was performed under four upper-limb postures: the arm relaxed naturally alongside the body, the arm elevated forward to approximately 45°, the arm elevated forward to approximately 90°, and the arm raised with the hand positioned in front of the eyes.

**Figure 1:**
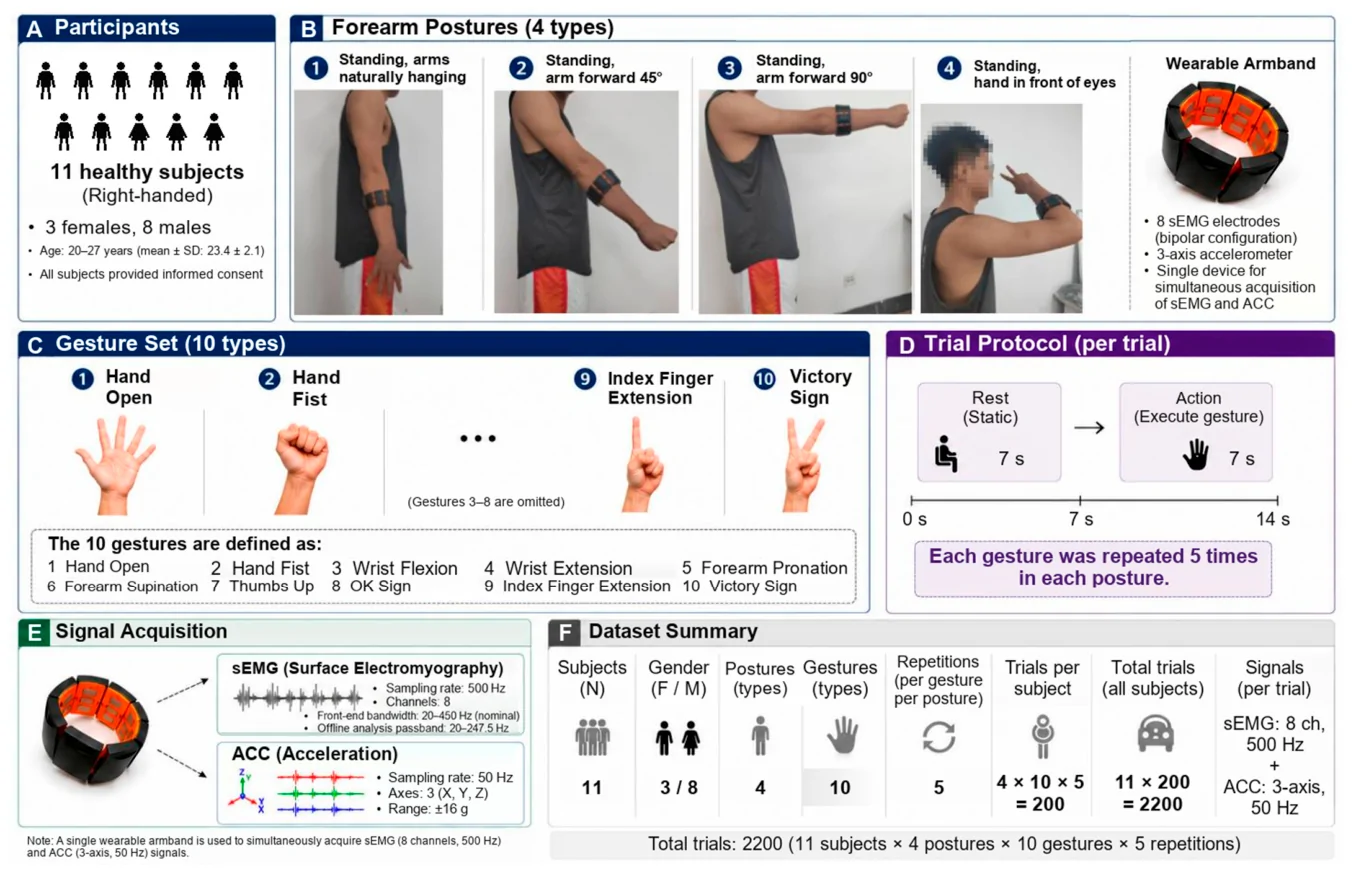
Overview of the multimodal sEMG–ACC gesture dataset. The figure presents participant information, forearm postures, gesture set, experimental procedure, signal acquisition parameters, and dataset scale. The 20–450 Hz value in panel E denotes the nominal device front-end bandwidth; all analyses used the offline effective passband of 20–247.5 Hz specified in Section 2.2.

One repetition was defined as one complete performance of the 40 gesture–posture combinations. Five repetitions were recorded from each participant, resulting in 200 action trials per participant and 2200 action trials across the complete dataset. Each trial comprised a 7 s resting phase followed by a 7 s gesture-execution phase. The planned acquisition was balanced across gesture classes, with 20 trials per gesture per participant and 220 trials per gesture across the cohort. Figure 1 summarizes the dataset and acquisition protocol.

### 2.2. Data Preprocessing and Window Construction

**Figure 2:**
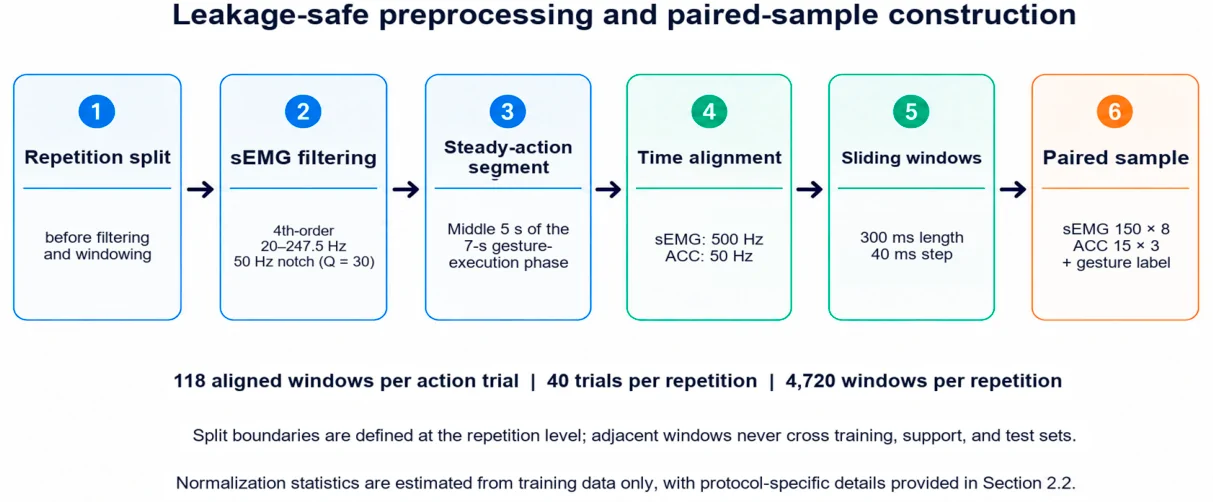
Leakage-safe preprocessing and sample construction. Repetition-level splitting precedes segmentation; each retained 5 s action segment yields 118 aligned windows. One repetition contains 40 trials (4720 windows), and normalization statistics are estimated from training data only (see Section 2.2).

Figure 2 summarizes preprocessing and window construction. The nominal 20–250 Hz sEMG band-pass was implemented using a Butterworth design with order 4 and cutoffs of 20 and 247.5 Hz: the requested upper cutoff was limited to 0.99 times the 250 Hz Nyquist frequency. Each channel was filtered forward and backward with the band-pass and then a 50 Hz notch filter (Q = 30), before central-segment extraction and windowing. This processing suppresses movement-related and power-line interference [6]. ACC signals were retained with the device-provided sampling and preprocessing configuration without additional offline temporal filtering. The same ACC preprocessing was used across the evaluated model comparisons.

For within-subject held-out evaluation, channel-wise z-score statistics were estimated exclusively from the four training repetitions of the current subject and applied unchanged to its held-out repetition. For cross-subject experiments, the statistics were estimated exclusively from source-subject pretraining samples and applied unchanged to source training, target support, and target test samples. In both protocols, statistics were computed after repetition-level splitting and window construction, before model input normalization, using x’ = (x − μ_c)/max(σ_c, 10^−4^). The middle 5 s of the 7 s gesture-execution phase of each trial was retained as the steady-action segment, excluding the movement onset and offset.

During sample construction, sliding windows were used to generate training samples from the steady-action segments. The sEMG window length was set to 150 sampling points, corresponding to about 300 ms, and the step size was set to 20 sampling points, corresponding to about 40 ms. ACC windows were aligned in time with the sEMG windows according to the sampling-rate ratio, thereby forming time-aligned sEMG-ACC sample pairs. Each sample contained one sEMG window, one aligned ACC window, and the corresponding gesture label. All training, adaptation, and test splits were defined at the repetition level before sliding-window segmentation, preventing adjacent windows from appearing in different subsets. Accordingly, one target repetition contains 200 s of retained signal (40 × 5 s) and 280 s of gesture execution (40 × 7 s). Including the prescribed 7 s rest phase in each trial, its scheduled recording time is 560 s (9 min 20 s), excluding device fitting, instructions, and other operational delays.

### 2.3. Problem Definition

In this paper, we consider simultaneously acquired surface electromyography (sEMG) signals and acceleration (ACC) signals. A sliding time-window strategy is adopted to segment the continuous signals. For each fixed-length time window, the model is required to learn discriminative temporal representations from the multimodal input and predict the corresponding gesture category. Formally, let the input of the i-th sample be denoted as:

(1) $$X_{i}^{\left(e\right)}\in R^{\left(C_{e}\times T_{e}\right)},X_{i}^{\left(a\right)}\in R^{\left(C_{a}\times T_{a}\right)}$$

where C_e_ and C_a_ denote the numbers of sEMG and ACC channels, respectively, and T_e_ and T_a_ denote their temporal lengths. In this study, $X_{i}^{\left(e\right)}\in R^{\left(8\times 150\right)}\;\mathrm{and}\;X_{i}^{\left(a\right)}\in R^{\left(3\times 15\right)}$ because the 300 ms windows are sampled at 500 Hz and 50 Hz, respectively. The corresponding gesture label is denoted as:

y_i_ ∈ {1,2,…,K} (2)

where K is the number of gesture classes. In this paper, we consider a multiclass gesture recognition task with K = 10.

**Figure 3:**
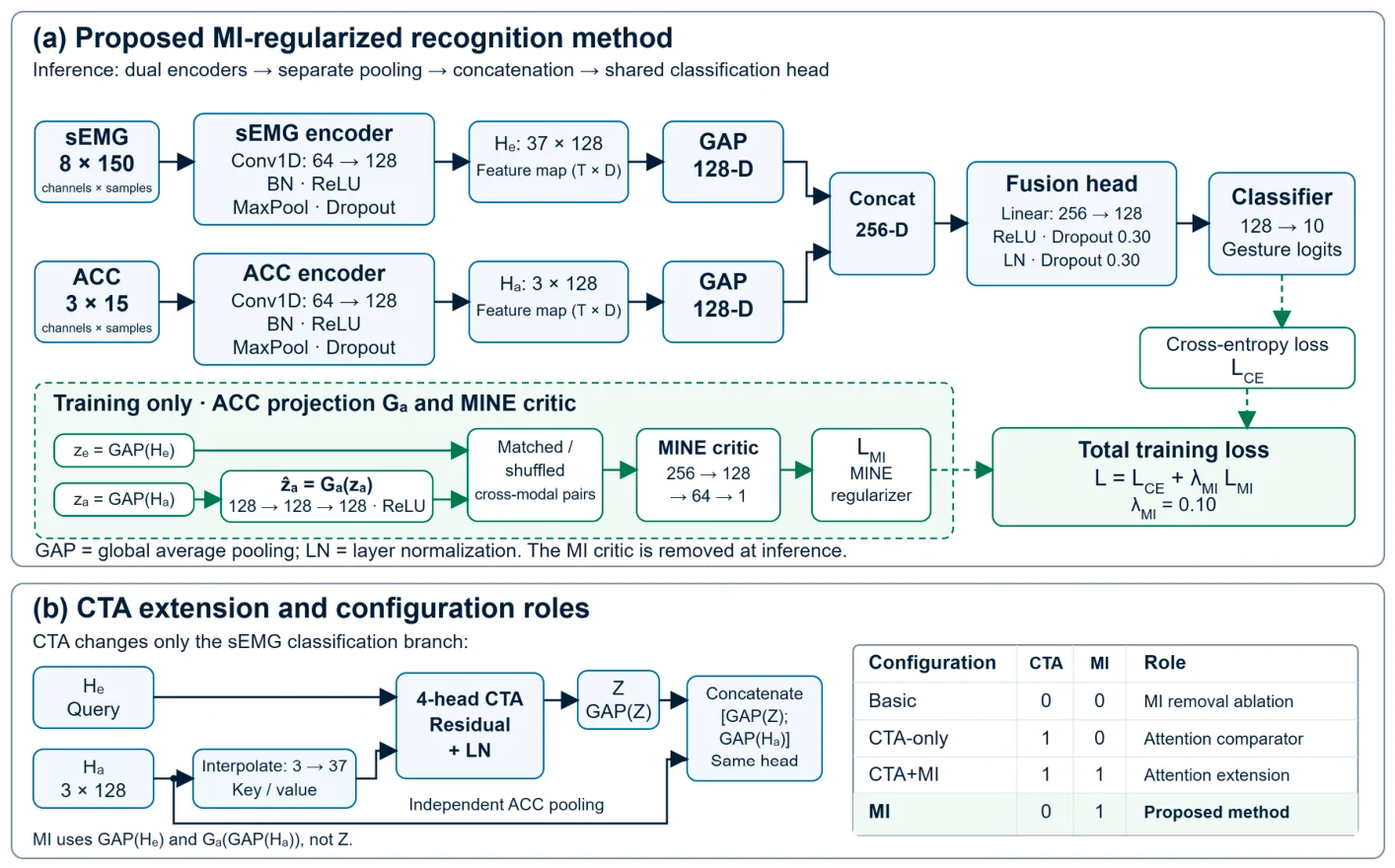
Proposed MI method and attention-based comparison configurations. (**a**) The classification path uses separate temporal encoders, global average pooling, concatenation, and classification. The training-only MI branch applies an ACC projection before the MINE critic; the projection and critic are omitted at inference. Raw inputs are labeled channels by samples, whereas encoded feature maps are labeled temporal positions by features, as in Equation (7). (**b**) CTA uses the sEMG sequence as queries and temporally aligned ACC features as keys and values. Its output updates the sEMG classification branch through residual addition and layer normalization; independent ACC pooling is retained. Square brackets and a semicolon denote concatenation. MI is computed from the encoder-derived representations rather than the CTA output.

The objective of this paper is to learn a parameterized discriminative function:

f(X^(e)^,X^(a)^;θ) (3)

where θ denotes the learnable parameters of the model. This function maps the multimodal temporal input to the prediction scores (or probability distribution) of the corresponding gesture classes. The overall framework is shown in Figure 3.

### 2.4. Dual-Branch Temporal Feature Extraction

The network adopts a dual-branch architecture to model the features of sEMG and ACC signals separately and performs information fusion in a high-level representation space. Each branch uses two unshared Conv1D blocks (kernel size 5, batch normalization, ReLU, Dropout1d 0.10, and max pooling), mapping sEMG from 8 channels to 128 features and ACC from 3 channels to 128 features. This design separates modality-specific temporal extraction from the later fusion and MI regularization stages.

Given a single-modal input signal X ∈ R^C×T^, feature encoding is performed through multiple layers of one-dimensional temporal convolutions, and the convolution operation at each layer can be expressed as:

H^(l)^ = σ(BN(Conv1D(H^(l−1)^))) (4)

where H^(l)^ denotes the feature map of the l-th layer, σ(·) denotes the ReLU activation function, and BN denotes batch normalization [29]. Each branch uses two Conv1D blocks with channel widths 64 and 128, kernel size 5, stride 1, same padding, followed by MaxPool1d(kernel size 2, stride 2) and Dropout1d(0.10). The encoder outputs are pooled separately for feature fusion in Section 2.5 and MI regularization in Section 2.6.

(5) $$H_{\mathrm{pool}}^{\left(l\right)}=\mathrm{MaxPool}\left(H^{\left(l\right)}\right)$$

Max pooling after each convolutional block downsamples the temporal sequence. The two modality encoders have independent parameters and are denoted as:

H^(e)^ = ℱ_ℯ_(X^(e)^), H^(a)^ = ℱ_𝒶_(X^(a)^) (6)

where ℱ_ℯ_(⋅)and ℱ_𝒶_(⋅) denote the temporal feature extraction functions for sEMG and ACC, respectively. Through the above encoding process, each modality is ultimately mapped into a high-level temporal feature sequence with a unified dimension.

(7) $$H^{\left(e\right)}\in R^{T_{e}^{\prime }\times D},H^{\left(a\right)}\in R^{T_{a}^{\prime }\times D}$$

This feature representation provides a structurally consistent input for subsequent global pooling, MI regularization, and feature fusion.

### 2.5. Feature Fusion and Classification

Figure 3a presents the proposed MI-regularized model. The two encoder sequences are globally averaged separately to obtain 128-dimensional sEMG and ACC vectors. These vectors are concatenated into a 256-dimensional representation and passed through Linear(256, 128), ReLU, Dropout(0.30), LayerNorm(128), feature Dropout(0.30), and Linear(128, 10). The final logits are used for supervised gesture classification. This inference path contains no attention module; MI regularization acts on the pooled encoder vectors during training, as described in Section 2.6.

The high-level temporal sequences are pooled independently and need not have equal encoded lengths. With encoded lengths T′_e_ and T′_a_, respectively, the pooled vectors are defined in Equation (8).

(8) $$z^{\left(e\right)}=\frac{1}{T_{e}^{\prime }}\sum_{t=1}^{T_{e}^{\prime }}H_{t}^{\left(e\right)},z^{\left(a\right)}=\frac{1}{T_{a}^{\prime }}\sum_{t=1}^{T_{a}^{\prime }}H_{t}^{\left(a\right)}$$

Both vectors are available to the classification head and to the training-only MI critic.

### 2.6. MI Regularization and Joint Training

The MI objective supplements supervised gesture classification by encouraging statistical dependence between the pooled modality-specific encoder representations. Both encoders receive gradients from the classification and MI objectives. The regularizer changes training but leaves the inference path in Section 2.5 unchanged.

Each modality contributes a 128-dimensional pooled encoder representation to the MI branch. Before the critic, the ACC vector is transformed by a training-only projection Gₐ consisting of Linear(128, 128), ReLU, and Linear(128, 128). The critic receives the concatenation of the pooled sEMG vector and the projected ACC vector. This projection is separate from the ACC vector used by the classification head and is omitted at inference.

We use a MINE-based regularizer [28] to encourage dependence between the sEMG and ACC representations alongside accurate gesture prediction. Shared information can also reflect posture, participant, or acquisition characteristics; the regularizer is therefore combined with classification supervision rather than treated as a guarantee of gesture-specific or subject-invariant features.

The Donsker–Varadhan objective underlying MINE [28] contrasts critic scores for joint pairs with an exponential term for shuffled pairs. The implemented objective in Equation (9) clips only the shuffled-pair scores before exponentiation. For a minibatch of N paired windows, let t_i_ be the critic score for the matched sEMG and projected ACC vectors, and let $\tilde{t_{i}}$ be the score after randomly permuting the projected ACC vectors within the minibatch.

(9) $$L_{\mathrm{MI}}=-\frac{1}{N}\sum_{i=1}^{N}t_{i}+\log(\frac{1}{N}\sum_{i=1}^{N}\exp(\min(\tilde{t_{i}},\;20))+\varepsilon )$$

The critic is a 256-128-64-1 multilayer perceptron with ReLU activations and dropout 0.20. In Equation (9), ε = 10^−8^ and the upper clipping threshold is 20; the joint-pair scores are not clipped. A random permutation supplies the marginal pairs without explicitly excluding fixed points. Because clipping is applied only to the marginal term, this MINE-based training surrogate is not interpreted as a certified Donsker–Varadhan lower bound on true MI. Joint training minimizes *L* = *L_CE_* + *λ_MI_L_MI_*, with an MI weight of 0.10. The MINE critic is optimized jointly with the encoders and classifier.

### 2.7. Cross-Subject Pretraining and One-Repetition Target Adaptation

**Figure 4:**
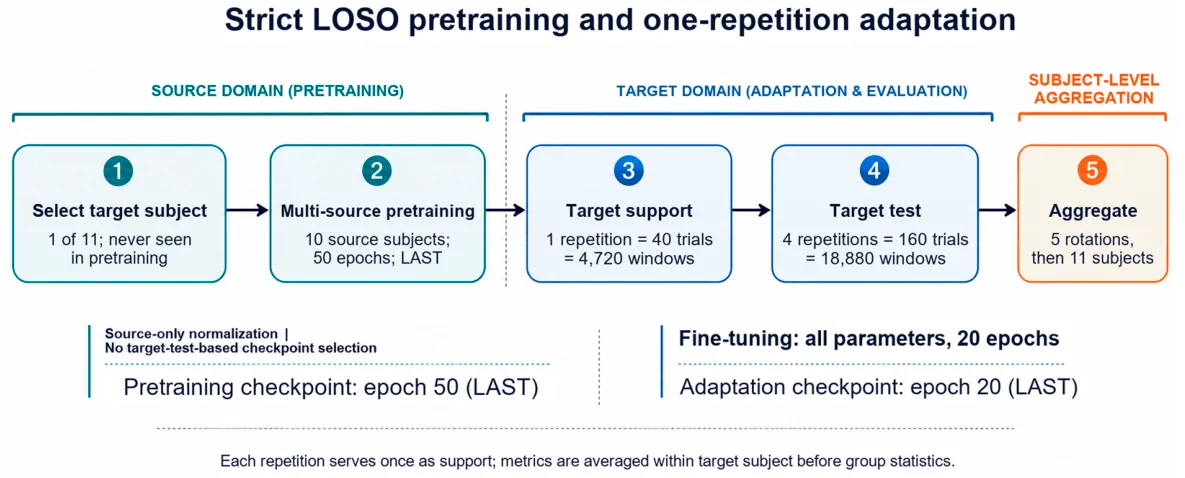
Strict LOSO pretraining and one-repetition adaptation protocol. The target subject is excluded from pretraining; final-epoch checkpoints are used at both stages; each of the five target repetitions serves once as support; and statistics are computed after within-subject fold aggregation.

A strict LOSO strategy is adopted, as summarized in Figure 4. In each round, one of 11 subjects is the target and the remaining 10 subjects are sources. The target is completely unseen during pretraining. Pretraining runs for 50 epochs and the epoch-50 LAST checkpoint is used. One of the target’s five repetitions (40 complete trials; 4720 windows) is then used for adaptation, while the other four repetitions (160 trials; 18,880 windows) are held out for testing. Adaptation runs for 20 epochs and the epoch-20 LAST checkpoint is used. Each support rotation is initialized independently from its corresponding source-pretrained state with a newly constructed optimizer; target-adapted parameters are not carried into the next rotation. In MI-enabled configurations, the pretrained critic is transferred with the recognition network and the MI term remains active during adaptation at the same weight of 0.10. This rotation is repeated five times per target subject; fold metrics are first averaged within target subject and then summarized across the 11 subjects.

To avoid leakage, normalization statistics are estimated only from source-subject training data and are applied unchanged to source training, target support, and target test data. Target-test samples are never used for normalization, gradient updates, early stopping, or per-fold checkpoint selection.

### 2.8. Ablation and Attention-Based Comparison Configurations

Basic fusion removes the MI term from the proposed method while retaining the same encoders, pooling, concatenation, and classification head. CTA-only provides an attention-based comparator trained with classification loss alone. CTA+MI adds the CTA operation to MI-regularized learning. Figure 3b summarizes these roles. The four configurations retain a complete 2 × 2 design, with c indicating the presence of CTA and m indicating the MI training objective: Basic fusion (0, 0), CTA-only (1, 0), CTA+MI (1, 1), and MI (0, 1).

Because the separately encoded sEMG and ACC feature sequences have different temporal lengths, the encoded ACC sequence is aligned to the encoded sEMG length only when c = 1. After the two max-pooling operations, the 150-sample sEMG input yields T′_e_ = 37 encoded positions, whereas the 15-sample ACC input yields T′ₐ = 3; CTA therefore interpolates the ACC sequence from 3 to 37 positions. Equation (11) defines one scaled dot-product attention head; the implemented CTA module uses four heads followed by concatenation and an output projection. The CTA output replaces only the sEMG sequence supplied to global pooling in the classification path. The MI branch retains the encoder-derived pooled vectors and the training-only ACC projection specified in Section 2.6.

The aligned ACC sequence is defined as

(10) $$\tilde{H^{\left(a\right)}}=\mathrm{Interp}\left(H^{\left(a\right)},T_{e}^{\prime }\right)$$

where Interp(·) denotes linear interpolation along the temporal dimension, H^(a)^ is the encoded ACC sequence, and T′_e_ is the encoded sEMG feature-sequence length. Thus, the aligned ACC representation $\tilde{H^{\left(a\right)}}$ has length T′_e_; T′_e_ is not the raw sEMG input length T_e_.

The CTA module treats sEMG features as Query and aligned ACC features as Key and Value to model temporal interactions:

(11) $$\mathrm{Attn}\left(Q,K,V\right)=\mathrm{softmax}\left(\frac{QK^{⊤}}{\sqrt{d_{k}}}\right)V$$

where Q, K, and V are the query, key, and value matrices. With h = 4 attention heads and total feature dimension D = 128, the per-head key dimension is d_k_ = D/h. The factor √d_k_ scales each dot product before softmax. The four head outputs are concatenated and projected by the multi-head module. The output is combined with the original sEMG sequence through a residual connection and layer normalization, as specified in Equation (12).

(12) $$Z=\mathrm{LayerNorm}\left(H^{\left(e\right)}+\mathrm{MultiHead}\left(H^{\left(e\right)},\tilde{H^{\left(a\right)}},\tilde{H^{\left(a\right)}}\right)\right)$$

Equation (12) therefore changes only the sEMG representation supplied to the shared fusion head: the classifier receives [GAP(Z); GAP(H^(a)^)] when CTA is enabled and [GAP(H^(e)^); GAP(H^(a)^)] otherwise. The ACC pooling path, fusion-head dimensions, and classifier are unchanged across CTA conditions.

Across the comparison configurations, the objective is *L*(*c*,*m*) = *L_CE_*(*c*) + *mλ_MI_L_MI_*, with the MI weight fixed at 0.10. In CTA+MI, MINE receives the pooled sEMG vector and the projected pooled ACC vector defined in Section 2.6; the encoders receive gradients from both the MI term and the CTA-conditioned classification path.

## 3. Experimental Setup

### 3.1. Evaluation Framework

Evaluation comprised within-subject testing and strict LOSO testing before and after one-repetition adaptation. Recognition performance was compared across the configurations defined in Section 2.8 and the baselines described in Section 3.3. An equal-budget experiment assessed source-subject composition with the MI model held fixed.

### 3.2. Evaluation Protocols

Three protocols are used. (1) Within-subject five-fold validation: one complete repetition is tested and the other four are trained, and every model is evaluated at its fixed final epoch. (2) Strict LOSO before adaptation: the target subject is excluded from pretraining, and the fixed epoch-50 LAST model is evaluated on the target test repetitions. (3) LOSO after one-repetition adaptation: one target repetition (4720 windows) is used as support and the other four (18,880 windows) as test data; all five rotations are evaluated with the epoch-20 LAST model. The support repetition comprised 40 gesture–posture trials, corresponding to 9 min 20 s of scheduled recording, as detailed in Section 2.2. Cross-subject metrics are averaged over the five rotations for each target before reporting the mean and sample standard deviation across 11 targets.

### 3.3. Comparison Methods and Evaluation Metrics

Comparisons have two roles. Configuration comparisons use the proposed MI model, its matched Basic fusion ablation, CTA-only, and CTA+MI as defined in Section 2.8. Other comparisons include ACC-only and sEMG-only inputs, CNN-LSTM within subjects, and MSCNN-TL [14] and an ACC-adapted Multistream CNN [15] for cross-subject transfer. MSCNN-TL uses sEMG with the local 8-channel, 150-sample, 10-class input/output settings. Its source pretraining combines gesture classification with a source-subject domain-adversarial loss of weight 0.5; the gradient-reversal scale follows 2/(1 + exp(−10p)) − 1, where *p* is the fraction of source optimization steps completed. During target adaptation, the multiscale branches and initial fusion block are parameter-frozen, while the deeper feature blocks and gesture head are updated. Batch-normalization running statistics continue to update from support batches, including in the parameter-frozen blocks; the domain-adversarial loss is disabled. The local Multistream CNN replaces the reference IMU input with three-axis ACC. It uses three sEMG temporal blocks (64, 128, and 128 channels), two ACC blocks (32 and 64 channels), separate global pooling, concatenation, a 128-unit fusion layer, and a ten-class classifier. Both streams and the fusion/classification head are fine-tuned with batch-normalization updates and no domain-adversarial loss. This ACC-adapted model is not an exact reproduction of the reference IMU implementation. All cross-subject comparisons use the same target splits and fixed epoch-50/epoch-20 LAST-to-LAST evaluation policy. Accuracy and Macro-F1 are reported throughout.

Table 1 summarizes the status of the comparison implementations.

The MI–Basic comparison isolates the added training objective with the same recognition backbone. Comparisons with the transfer-learning baselines concern their evaluated local implementations under the shared protocol.

For statistical inference, the participant is the independent sampling unit. Accuracy and Macro-F1 are computed at window level; fold metrics are first averaged within participant. The 300 ms windows with a 40 ms step are strongly correlated, so 4720 windows do not represent 4720 independent observations. The analysis includes 11 participants; no trial-level voting is applied. Primary recognition tables report participant-level mean ± sample SD (ddof = 1; *n* = 11); descriptive figures and auxiliary intervention summaries display means where specified.

Model-to-baseline tests use Holm adjustment across five paired comparisons separately for each stage and metric. Friedman tests compare the four configurations, followed by paired Wilcoxon signed-rank tests. Within subjects, Holm adjustment covers all six configuration pairs separately for each metric. The MI-focused cross-subject comparisons are exploratory analyses of the configuration results, with attention directed to MI following its highest observed mean performance. Within each cross-subject stage and metric, Holm correction covers its comparisons with Basic fusion, CTA-only, and CTA+MI. The same MI–Basic contrast can therefore have different adjusted *p* values in the configuration and model-to-baseline families. These families are retained throughout the results.

For gesture-wise evaluation, classification sensitivity is defined as recall, TP/(TP + FN), and F1 combines precision and recall. The five support-fold metrics are averaged within each target participant before reporting the mean and sample SD across 11 participants. Mean row-normalized confusion matrices use the same fold-to-participant aggregation. Classification recall is distinct from hardware sensor sensitivity or resolution and from clinical diagnostic sensitivity; the latter properties were not evaluated here.

For posture-wise evaluation, held-out predictions from Basic fusion and MI were grouped by the four arm postures defined in Section 2.1. Accuracy and ten-class Macro-F1 were calculated separately for each participant, support fold, posture, and adaptation stage. The five-fold values were averaged within each participant and then summarized as mean ± sample SD across the 11 target participants. Within each fold, the two models and the before/after-adaptation evaluations used the same test windows. Posture-wise results were reported descriptively.

The participant-level interaction contrast is Δint = (CTA+MI − MI) − (CTA-only − Basic). Exact two-sided Wilcoxon tests compare the 11 participant-level interaction contrasts with zero, with Holm adjustment across three settings and two metrics. The interaction analysis uses the same participant-level summaries as the configuration comparison. The source-diversity experiment uses Holm adjustment across its four outcomes. The auxiliary analyses use three separate Holm families: 22 tests for cross-modal similarity diagnostics, six tests for class-scatter ratios, and eight tests for attention-strength interventions, as specified in Section 3.6. Prediction-error comparisons are descriptive. Statistical significance is defined as adjusted *p* < 0.05.

### 3.4. Implementation Details

The models were implemented using PyTorch 2.11.0 (CUDA 12.8 build) and trained on an NVIDIA GeForce RTX 3090 GPU, with a feature dimension of 128 and the two-block Conv1D encoders described in Section 2.4. The proposed MI method and Basic fusion use the same fusion head: Linear(256, 128)-ReLU-Dropout(0.30)-LayerNorm(128)-Dropout(0.30)-Linear(128, 10). The attention configurations retain this head after the four-head CTA update defined in Section 2.8. Adam [30] is used with a pretraining learning rate of 5 × 10^−4^, weight decay of 1 × 10^−4^, batch size 128, and 50 epochs. In MI-enabled configurations, the MINE critic is optimized jointly with the classifier using the same Adam optimizer and learning rate. Adaptation uses a learning rate of 2 × 10^−4^ for 20 epochs, with all parameters updated. Encoder, CTA, and classifier dropout rates are 0.10, 0.20, and 0.30, respectively; label smoothing is 0.05. Gradients are clipped to a maximum norm of 5.0. All experiments use seed 42 with deterministic cuDNN settings where available. The MI critic is training-only, whereas CTA remains part of inference in the attention configurations.

For the CNN–LSTM baseline, the same preprocessed sEMG–ACC windows, repetition-level five-fold split, normalization procedure, and subject-level aggregation were used as for the evaluated CTA/MI variants. The sEMG branch consisted of two temporal convolutional blocks with 64 hidden and output channels, whereas the ACC branch used 32 hidden channels and 64 output channels. Each block consisted of a one-dimensional convolution with kernel size 5 and padding 2, followed by batch normalization, ReLU activation, and max pooling with kernel size 2. The two 64-dimensional modality sequences were temporally aligned by linear interpolation when necessary and concatenated into a 128-dimensional sequence. A dropout layer with a rate of 0.10 was applied before a single-layer unidirectional LSTM with a hidden size of 128. The LSTM outputs were temporally averaged and passed through a classifier consisting of Linear(128, 128), ReLU, Dropout(0.20), and Linear(128, 10). With eight sEMG channels and three ACC channels, the baseline contained 184,330 trainable parameters. The model was trained for 50 epochs using Adam with a learning rate of 5 × 10^−4^, weight decay of 1 × 10^−4^, and a batch size of 128. All experiments used random seed 42 and the fixed final-epoch (LAST) checkpoint; no validation-set selection, early stopping, or test-based checkpoint selection was performed.

No validation set was used in the cross-subject experiments. The 50-epoch pretraining and 20-epoch adaptation budgets were determined from preliminary observations of training stability and kept fixed across participants and folds. Evaluation used the final checkpoint at each stage, and all within-subject models were likewise evaluated at their fixed final epoch. Target-test performance was not used for early stopping, checkpoint selection, or selection of the epoch budgets. The remaining CTA and MI hyperparameters were held fixed across participants and folds.

Computational cost was evaluated for the proposed MI model’s classification inference path after excluding the training-only ACC projection and MINE critic. Inputs consisted of one 300 ms sEMG window of shape [1, 8, 150] and the corresponding ACC window of shape [1, 3, 15]. Inference parameter counts included all retained network parameters and excluded non-parameter buffers. MAC-based FLOPs were calculated as twice the convolutional and linear-layer multiply–accumulate count, with one multiplication and one addition counted as two floating-point operations. Bias additions, normalization, activation, pooling, and other scalar operations were excluded from this MAC-based measure.

Host-CPU inference latency was measured on an Intel Core Ultra 9 275HX running Windows 11 build 26200 with PyTorch 2.11.0. Measurements used FP32, one CPU thread, and a batch size of one, with the model in evaluation mode and gradient computation disabled through inference mode. Following 100 warm-up passes, five groups of 1000 forward passes were timed using preloaded inputs without profiling. Timing excluded model loading, data reading, input generation, preprocessing, loss computation, and evaluation calculations. Mean latency and sample SD were calculated over all 5000 timed passes.

### 3.5. Sensitivity to the MI Regularization Weight

During model development, a preliminary sensitivity analysis of the MI regularization weight was conducted under the strict LOSO protocol before target adaptation. The model architecture, preprocessing, optimizer, training schedule, and all other settings were held fixed; only λ was changed. Table 2 reports this preliminary analysis, with λ = 0 corresponding to the matched Basic setting without MI regularization.

Moderate nonzero MI weights improved recognition relative to λ = 0, and λ = 0.05–0.10 yielded similar performance. Performance declined slightly at λ = 0.20. Based on this preliminary comparison, λ = 0.10 was adopted as a practical setting for subsequent experiments, rather than a statistically established optimum.

### 3.6. Auxiliary Representation and Prediction Diagnostics

Post-training diagnostics examined representation and prediction differences among CTA-only, MI, and CTA+MI on held-out data without updating model parameters. The analyses covered cross-modal similarity, class geometry, prediction differences, and sensitivity to the attention update.

#### 3.6.1. Cross-Modal Similarity Diagnostics

Within-subject cross-modal similarity diagnostics characterized CTA-only, MI, and CTA+MI on held-out repetitions. The cross-modal similarity analysis covered 11 participants and five folds per model, using all 4720 test windows in each participant–fold combination. Feature extraction used evaluation mode without parameter updates, and diagnostic values were averaged across the five folds within each participant. Basic fusion was not included in these representation diagnostics, so they characterize the attention-related comparisons rather than directly isolating the effect of adding MI to Basic fusion.

Cross-modal representational similarity was quantified using linear centered kernel alignment (CKA) [31]. For sample-by-feature matrices X and Y whose columns were centered across test windows, CKA was calculated as the squared Frobenius norm of XᵀY divided by the product of the Frobenius norms of XᵀX and YᵀY. Higher CKA indicates greater similarity in representation structure; it is not an estimate of mutual information or class-discriminative content. The sEMG reference was the globally pooled encoder sequence. For the attention configurations, the ACC reference was averaged after linear temporal interpolation from 3 to 37 positions; for MI, it was averaged at the encoder’s native temporal resolution. Cross-configuration CKA contrasts therefore characterize these extracted representations and are not attributed exclusively to learned encoder differences. Pre-/post-attention comparisons within each attention configuration used the same ACC reference.

For CTA-only and CTA+MI, the change after attention fusion was defined as CKA between the pooled CTA output and the ACC reference minus CKA between the pooled sEMG encoder output and the same ACC reference. Thus, this measure includes both the residual attention contribution and layer normalization. The relative attention contribution was measured for each window as the Frobenius norm of the multi-head attention output, after its output projection and before residual addition, divided by the Frobenius norm of the sEMG query sequence; window-level ratios were averaged within each fold.

Secondary Procrustes similarity was computed as the sum of singular values of the cross-product between column-centered feature matrices after separate Frobenius normalization. Its change after attention fusion used the same post-fusion-minus-reference definition as the CKA change. Projection-space metrics compared the pooled sEMG reference with Gₐ applied to the ACC reference defined above, using the learned Linear(128, 128)–ReLU–Linear(128, 128) mapping. The fusion Fisher ratio was the trace of between-class scatter divided by the trace of within-class scatter for the pooled representation entering the classifier.

Exact two-sided Wilcoxon signed-rank tests used the 11 participant-level five-fold means. Holm adjustment was applied jointly to a single family of 22 tests: all three pairwise comparisons among CTA-only, MI, and CTA+MI for accuracy, Macro-F1, encoder-level CKA, and encoder-level Procrustes similarity (12 tests); changes in CKA and Procrustes similarity tested against zero in each attention-based model and compared between these models (6 tests); CTA-only versus CTA+MI comparisons of the attention-output/query norm ratio and fusion Fisher ratio (2 tests); and MI-versus-CTA+MI comparisons of CKA and Procrustes similarity in the projection space defined above (2 tests). The main diagnostics are encoder-level CKA, its change after attention fusion, and the attention-output/query norm ratio.

#### 3.6.2. Class Geometry and Prediction Differences

A further analysis examined CTA-only, MI, and CTA+MI within subjects and after cross-subject adaptation, covering 11 participants and five folds in each setting. Saved labels and predictions were used to identify the corresponding evaluation records. This analysis was conducted separately from the CKA analysis in Section 3.6.1. All measurements used held-out repetitions.

Class geometry was measured from the activation supplied directly to each model’s final gesture-classification linear layer, captured before multiplication by that layer’s weight matrix and after all preceding feature transformations. Each sample feature was normalized to unit L2 norm. Within-class scatter W was the mean squared distance from each normalized feature to its class centroid. Between-class scatter B was the sample-count-weighted mean squared distance from class centroids to the overall centroid. The ratio R = W/B summarizes relative within-class dispersion. Fold values were averaged within participant, and exact two-sided Wilcoxon tests compared all three model pairs in both settings, with Holm adjustment across these six R comparisons. W and B were reported descriptively.

Prediction differences were calculated from the original saved predictions on identical test windows. For each model pair, we measured the percentage of all test windows correctly classified by only one model and the percentage misclassified by both. The difference between the two exclusive-correct percentages equals their accuracy difference in percentage points. These quantities were averaged across folds within participant and then across participants. They describe output differences and were not subjected to additional significance tests.

#### 3.6.3. Fixed-Weight Attention Interventions

For the CTA-only and CTA+MI models in Section 3.6.2, the multi-head attention update was multiplied by a fixed scalar alpha after its output projection and before residual addition to the sEMG sequence and layer normalization. We evaluated alpha = 0, 0.5, and 1, corresponding to suppression, halving, and the unaltered update. Only this attention-update tensor was scaled; the encoder outputs and all remaining forward operations were left unchanged. All encoder, attention, normalization, and classifier weights remained fixed. Setting alpha = 0 is not equivalent to a separately trained MI model or a retrained attention ablation.

Each of the 220 model–participant–fold combinations was evaluated at all three strengths. The unaltered-model predictions agreed with the saved predictions by at least 99.9788% in every fold; intervention differences used the recomputed alpha = 1 predictions as their paired reference. Accuracy was the primary endpoint. Exact two-sided Wilcoxon tests compared alpha = 0 and 0.5 with alpha = 1 in each model and setting, with Holm adjustment across eight tests. Macro-F1 and prediction switches were descriptive. The three strengths and test family were fixed before the intervention run, and no strength was selected to replace the original recognition results.

## 4. Results

### 4.1. Gesture-Wise Recognition Before and After One-Repetition Adaptation

**Figure 5:**
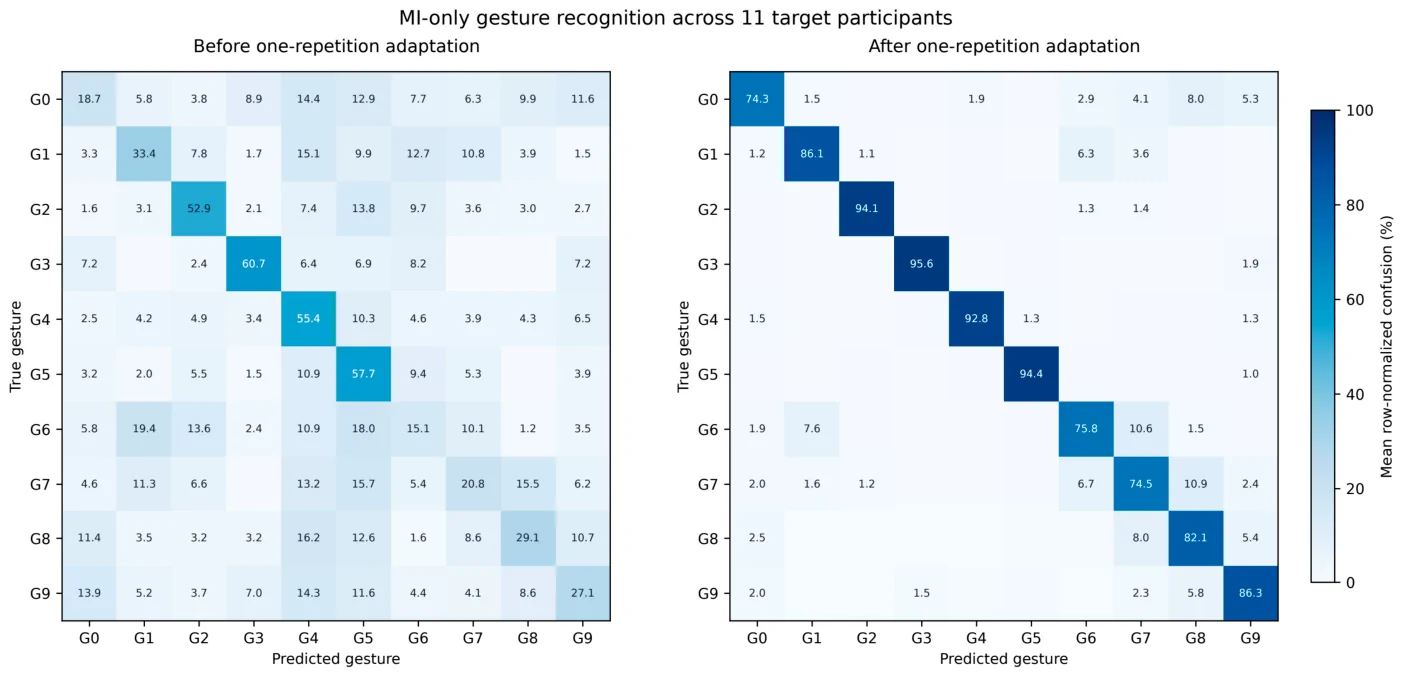
Mean row-normalized confusion matrices for the MI model before and after one-repetition adaptation. Values are percentages across 11 target participants, after averaging the five support folds within each participant. Rows denote true gestures and columns predicted gestures; gesture codes are defined in Table 3.

Figure 5 and Table 3 present the MI model’s gesture-wise recognition before and after one-repetition adaptation across 11 target participants. The class-wise analysis shows that the improvement after adaptation is distributed across the gesture set rather than being driven by a single class. After adaptation, wrist extension (G3), forearm supination (G5), and wrist flexion (G2) achieved recalls of approximately 95.6%, 94.4%, and 94.1%, respectively. Hand opening (G0), thumbs-up (G6), and the OK sign (G7) remained more difficult, with recalls of 74.3%, 75.8%, and 74.5%. These results characterize the class-wise effect of target adaptation within the MI model.

### 4.2. Cross-Subject Recognition Performance

#### 4.2.1. Before Target Adaptation

**Figure 6:**
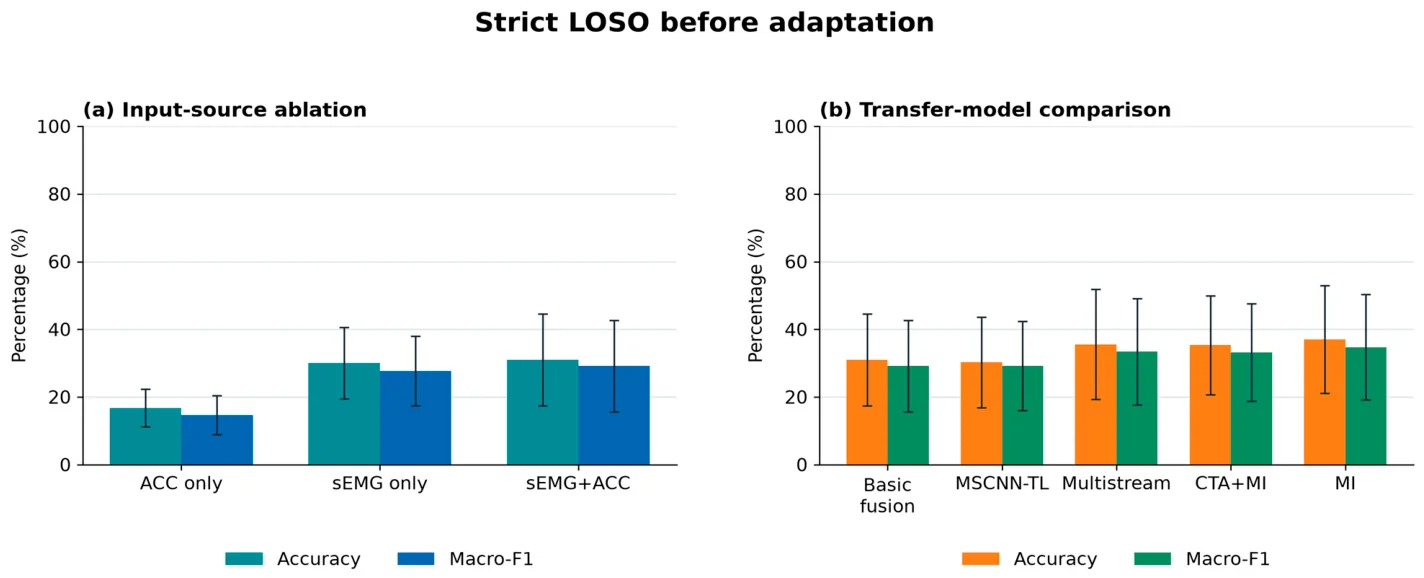
Strict LOSO results before adaptation under the fixed epoch-50 LAST policy. Bars show means and error bars show participant-level sample SD across 11 target participants; Table 4 reports means and sample SD for both panels. (**a**) Input-source ablation; (**b**) transfer-model comparison including CTA+MI and MI. CNN-LSTM is not included because only its within-subject result was available.

Figure 6 reports strict LOSO recognition before target adaptation. MI achieves 37.02% accuracy and 34.72% Macro-F1, compared with 31.02%/29.15% for Basic fusion, 30.24%/29.19% for MSCNN-TL, and 35.56%/33.40% for the ACC-adapted Multistream CNN. ACC-only and sEMG-only inputs yield 16.76%/14.63% and 30.00%/27.74%, respectively. Table 4 provides the transfer-model means and sample standard deviations across 11 target subjects. Direct transfer remains difficult for all evaluated methods; the attention configuration comparisons are detailed in Section 4.4.

Subject-level paired inference was conducted after averaging the five rotations within each target. With Holm correction across five baselines, MI was significantly better than ACC-only (adjusted *p* = 0.00488 for both metrics) and Basic fusion (adjusted *p* = 0.00488 for accuracy and 0.01172 for Macro-F1) before adaptation. The comparisons with sEMG-only, MSCNN-TL, and the ACC-adapted Multistream CNN did not remain significant after correction (adjusted *p* ≥ 0.07324).

#### 4.2.2. After One-Repetition Target Adaptation

**Figure 7:**
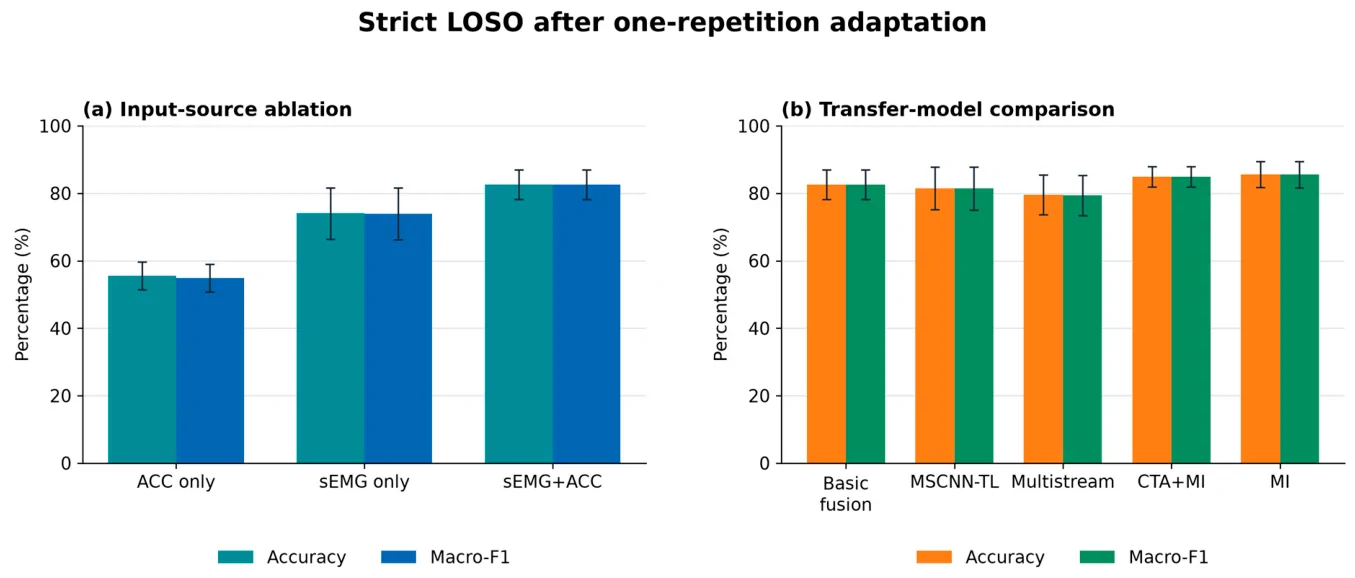
Strict LOSO results after one-repetition adaptation under the fixed epoch-20 LAST policy. Bars show means and error bars show participant-level sample SD across 11 target participants; Table 4 reports means and sample SD for both panels. (**a**) Input-source ablation; (**b**) transfer-model comparison including CTA+MI and MI.

After one-repetition target adaptation, MI reaches 85.61% accuracy and 85.57% Macro-F1 (Figure 7). The corresponding results are 82.55%/82.52% for Basic fusion, 81.50%/81.42% for MSCNN-TL, and 79.53%/79.36% for the ACC-adapted Multistream CNN. Relative to its own performance before adaptation, MI gains 48.59 percentage points in accuracy and 50.86 points in Macro-F1 (calculated from unrounded means). These changes describe the effect of target adaptation; the matched contribution of MI regularization is evaluated separately in Section 4.3. All methods use identical target support/test splits and the fixed LAST-to-LAST checkpoint policy. Table 4 reports participant-level mean ± sample SD.

After one-repetition adaptation, subject-level paired tests with Holm correction showed that MI exceeded ACC-only, sEMG-only, Basic fusion, and both transfer baselines for accuracy and Macro-F1 (all adjusted *p* ≤ 0.010).

#### 4.2.3. Posture-Wise Recognition Performance

Table 5 reports recognition performance separately for the four recorded arm postures. MI achieved higher mean accuracy and Macro-F1 than Basic fusion in each posture, both before and after one-repetition adaptation. The largest mean accuracy gain before adaptation occurred at 90° forward elevation (8.75 percentage points), whereas the largest gain after adaptation occurred with the arm relaxed alongside the body (4.02 points). After adaptation, MI accuracy ranged from 84.09% to 86.52%. The hand-in-front-of-eyes posture had the lowest MI mean performance at both stages.

### 4.3. Contribution of MI Regularization

#### 4.3.1. Matched Ablation Across Evaluation Settings

Table 6 summarizes the matched MI-versus-Basic ablation. Adding MI regularization raises mean Accuracy from 31.02% to 37.02% before cross-subject adaptation, from 82.55% to 85.61% afterward, and from 91.26% to 93.78% within subjects. The corresponding gains are 6.00, 3.06, and 2.52 percentage points. Macro-F1 increases from 29.15% to 34.72%, from 82.52% to 85.57%, and from 91.17% to 93.74%, respectively. These comparisons hold the inference architecture unchanged and directly assess the additional training objective.

The matched MI–Basic comparisons showed significant improvements in both accuracy and Macro-F1 across all three evaluation settings after Holm correction (Table 6). Complete four-configuration comparisons are reported in Section 4.4.

#### 4.3.2. Within-Subject Input and Architecture Comparisons

**Figure 8:**
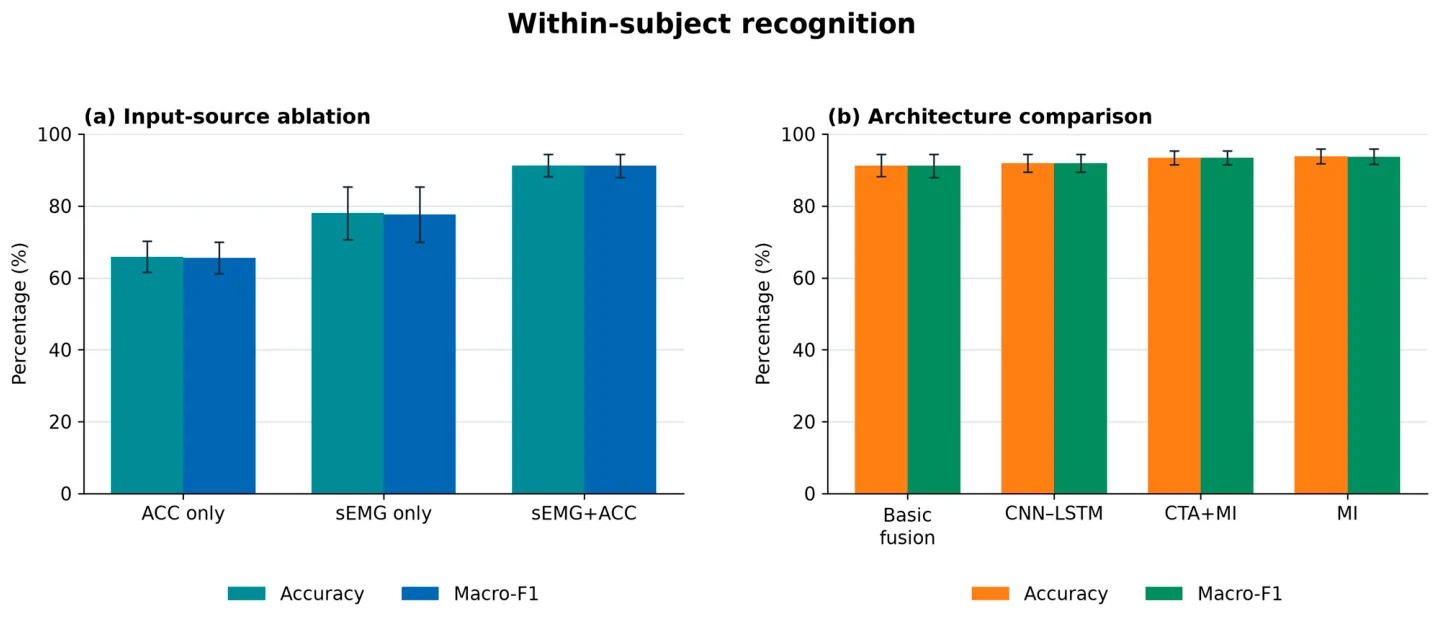
Within-subject results using fixed final-epoch checkpoints. (**a**) Input-source ablation; (**b**) architecture comparison. Accuracy and Macro-F1 are shown as percentages. All error bars represent participant-level SD across 11 participants.

Figure 8 separates the input-source ablation from the architecture comparison. ACC-only obtains 65.87% accuracy and 65.53% Macro-F1, sEMG-only obtains 78.03% and 77.62%, and Basic fusion reaches 91.26% and 91.17%. These results show that sEMG is the stronger individual source and that ACC provides complementary information when fused with sEMG.

In the architecture panel, CNN-LSTM reaches 91.90% accuracy and 91.87% Macro-F1. MI achieves 93.78% accuracy and 93.74% Macro-F1, exceeding CNN-LSTM by 1.88 and 1.87 percentage points and Basic fusion by 2.52 and 2.57 points, respectively.

### 4.4. Attention Extensions and Interaction Analysis

#### 4.4.1. Within-Subject Configuration Comparisons

**Figure 9:**
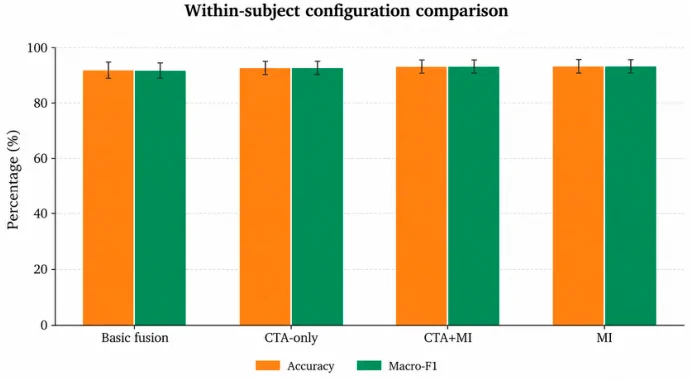
Complete within-subject configuration comparison using fixed final-epoch checkpoints. Accuracy and Macro-F1 are shown as percentages. Basic fusion removes MI from the proposed method; CTA-only and CTA+MI provide the attention comparator and extension, respectively. All error bars represent participant-level SD across 11 participants.

Figure 9 shows the complete within-subject configuration comparison. Basic fusion and MI assess the training-objective ablation, while CTA-only and CTA+MI examine attention without and with MI regularization.

CTA-only achieves 92.25% accuracy and 92.19% Macro-F1, and CTA+MI achieves 93.41% and 93.36%, respectively. Both exceed Basic fusion in mean performance but remain below MI, which achieves 93.78%/93.74%.

Friedman tests on subject-level averages indicated significant differences among the four within-subject variants for accuracy and Macro-F1 (χ^2^(3) ≈ 27.33, *p* = 5.03 × 10^−6^ for each metric). Holm-adjusted post hoc paired Wilcoxon tests across all six pairwise comparisons showed that all three CTA- and/or MI-enabled variants outperformed Basic fusion: CTA-only at adjusted *p* = 0.02734, and MI and CTA+MI at adjusted *p* = 0.00586 for each metric. MI outperformed CTA-only (adjusted *p* = 0.00586), CTA+MI outperformed CTA-only (adjusted *p* = 0.02051), and MI also outperformed CTA+MI (adjusted *p* = 0.02734).

#### 4.4.2. Cross-Subject Attention Comparisons

Table 7 reports all four configurations under the same strict LOSO pretraining and one-repetition adaptation protocol. Values are participant-level means and sample standard deviations after averaging the five folds within each target.

CTA-only achieves 35.23%/32.91% accuracy/Macro-F1 before adaptation and 84.81%/84.86% afterward. The corresponding CTA+MI results are 35.32%/33.20% and 84.91%/84.92%. MI has the highest observed means at both stages.

Friedman tests on the participant-level summaries indicate differences among the four configurations: χ^2^(3) = 23.836 (*p* = 2.70 × 10^−5^) for accuracy before adaptation, χ^2^(3) = 15.545 (*p* = 0.00141) for Macro-F1 before adaptation, χ^2^(3) = 17.945 (*p* = 0.000451) for accuracy after adaptation, and χ^2^(3) = 19.364 (*p* = 0.000230) for Macro-F1 after adaptation. The MI–Basic comparisons are reported in Section 4.3.1.

CTA+MI did not exceed MI in observed mean performance at either cross-subject stage. However, the MI differences from CTA-only and CTA+MI were not significant after the three-comparison Holm correction (adjusted *p* ≥ 0.06445 before adaptation and ≥0.13477 afterward). Therefore, under the evaluated conditions, CTA did not provide additional mean performance improvement over MI. Further investigation is required to determine when attention-based fusion provides complementary benefits.

#### 4.4.3. CTA × MI Interaction Analysis

The interaction contrast defined in Section 3.3 compares the CTA+MI-minus-MI difference with the CTA-only-minus-Basic difference. Negative values indicate a smaller attention-associated performance difference when MI is enabled. This test of departure from an additive pattern is distinct from the direct MI-versus-CTA+MI comparison.

Within subjects, the interaction contrast was negative for 8 of 11 participants for accuracy and 9 of 11 participants for Macro-F1, with mean contrasts of −1.35 ± 1.25 and −1.39 ± 1.32 percentage points, respectively. The exact two-sided Wilcoxon *p* values were 0.01367 and 0.00977, respectively; both remained significant after Holm correction (adjusted *p* = 0.01953). Before cross-subject adaptation, the contrast was negative for all 11 participants and was −5.91 ± 3.50 points for accuracy and −5.28 ± 3.77 points for Macro-F1. After adaptation, it was negative for all 11 participants and was −2.96 ± 1.23 and −2.99 ± 1.22 points, respectively. The exact two-sided Wilcoxon *p* value was 0.00098 for each of the four cross-subject outcomes, and all four remained significant after Holm correction (adjusted *p* = 0.00586). Thus, all six stage-by-metric interaction tests remained significant after correction.

The negative contrasts indicate a consistent sub-additive performance pattern across within-subject recognition and both cross-subject stages.

### 4.5. Source-Subject Diversity Under an Equal Data Budget

An equal-budget experiment evaluated source-subject composition with the MI model held fixed. Each condition contains 200 complete source trials (23,600 windows). For each target, ten paired source plans were generated from a fixed-seed ordering of the other ten participants. For target index s (zero-based), this ordering used NumPy default_rng with seed 42 + 1009 × *s*. In plan *p* = 0, …, 9, the single-source condition used all five repetitions of participant *p* in that ordering. The multi-source condition used participants at cyclic positions (*p* + j) modulo 10, j = 0, …, 4, with repetition (2j + *p*) modulo 5 from each participant. Each multi-source plan therefore contained all five repetition indices once; across ten plans, every source participant contributed each repetition once. Whole subject–repetition blocks were used without window-level subsampling.

Each plan was evaluated over five target-support rotations, each restarting from that plan’s epoch-50 source checkpoint, including its critic parameters, with a fresh optimizer. The MI weight remained 0.10 during adaptation. The 50 plan-by-rotation results were averaged within target before means, sample SD, and paired tests were calculated across the 11 targets. Source plans were not selected using target-test performance.

**Figure 10:**
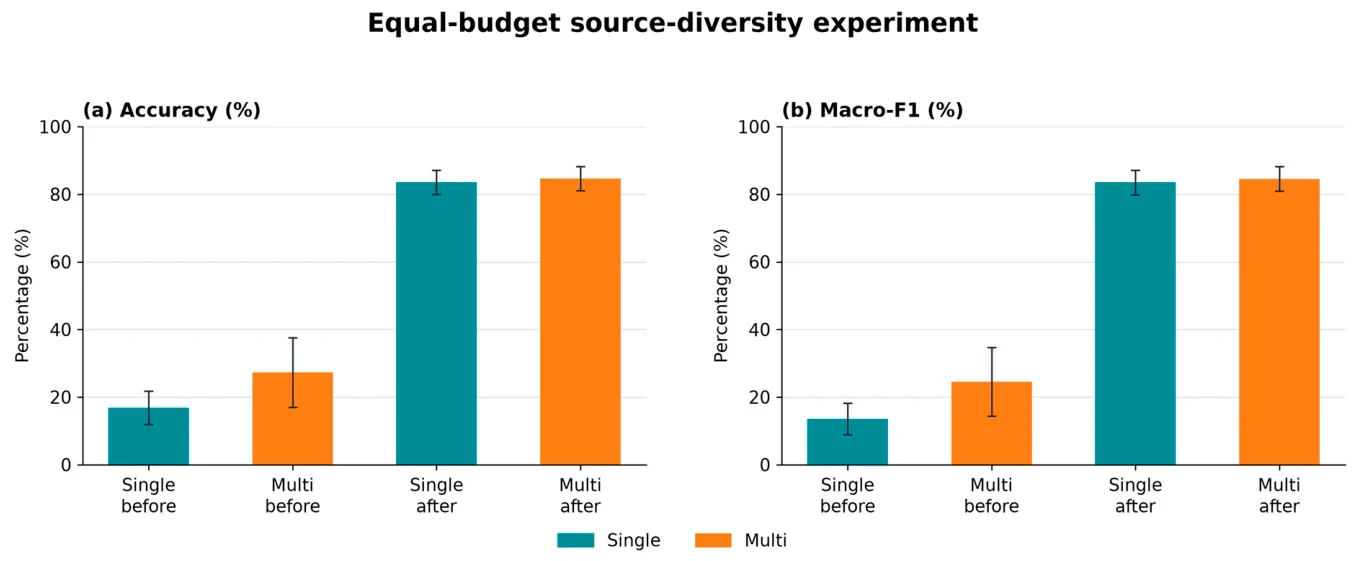
Equal-budget source-composition results for the MI configuration. Both source conditions use 200 trials (23,600 windows); bars show means and error bars show the sample SD across 11 target-participant means, after averaging source plans and support folds within each participant (ddof = 1).

Figure 10 shows that, before adaptation, single-source pretraining yields 16.84% accuracy and 13.59% Macro-F1, whereas multi-source pretraining yields 27.27% and 24.48%, gains of 10.43 and 10.89 percentage points. After adaptation, the corresponding results are 83.53%/83.47% and 84.62%/84.56%, a smaller but consistent gain of 1.09 points in both metrics.

Paired Wilcoxon tests with Holm correction confirm the multi-source advantage before adaptation (adjusted *p* = 0.00586 for accuracy and 0.00391 for Macro-F1).

After adaptation, the multi-source condition also shows a significant advantage under the matched design (Holm-adjusted *p* = 0.00586 for both accuracy and Macro-F1). Equal sample count does not completely exclude the influence of source identity, signal quality, or other source characteristics.

### 4.6. Auxiliary Representation and Prediction Analyses

#### 4.6.1. Cross-Modal Representation Similarity

We examined whether differences in cross-modal similarity, class geometry, and reliance on the attention update were accompanied by corresponding differences in recognition performance.

**Figure 11:**
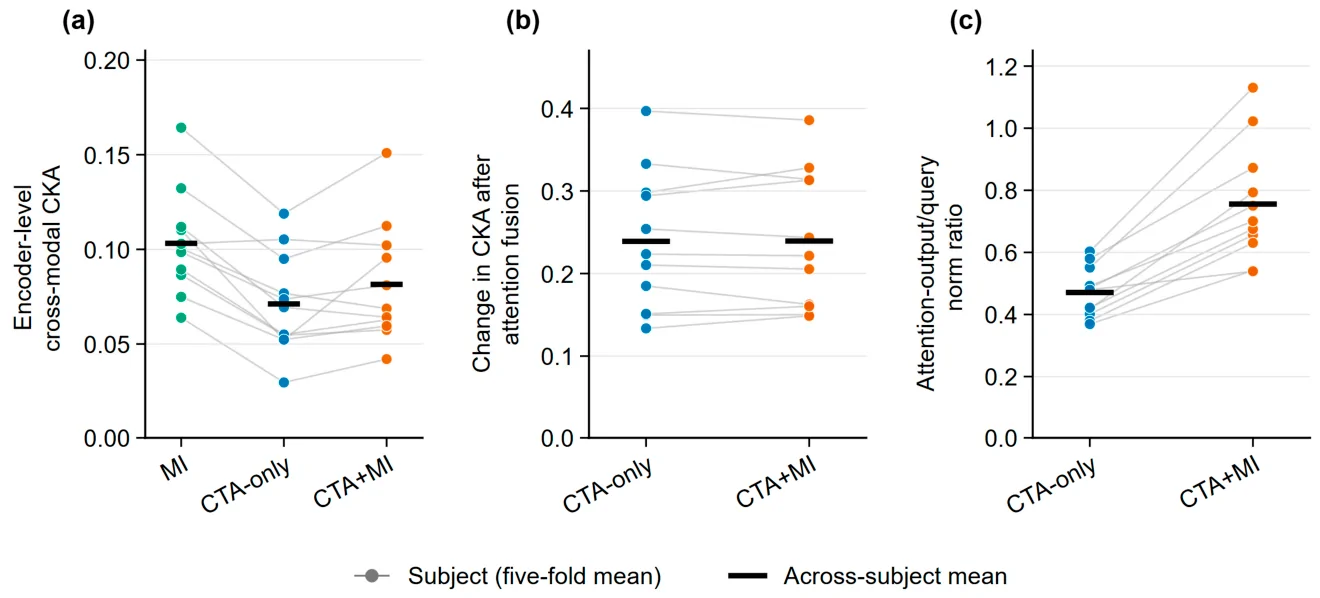
Auxiliary within-subject representation diagnostics. (**a**) Cross-modal CKA for MI, CTA-only, and CTA+MI, using the ACC reference extraction rules in Section 3.6.1. (**b**) Change in CKA after attention fusion relative to the pooled sEMG encoder representation, with the same ACC reference retained for each pre/post comparison. (**c**) Attention-output/query norm ratio before residual addition and layer normalization. Each colored point is one participant’s five-fold mean (*n* = 11); gray lines connect the same participant across models, and black horizontal lines indicate group means. Panel (**b**) measures representational similarity after multimodal mixing; panel (**c**) measures the relative magnitude of the attention branch.

Figure 11 summarizes the within-subject representation analysis. Cross-modal CKA was 0.1032 ± 0.0275 for MI, 0.0712 ± 0.0265 for CTA-only, and 0.0815 ± 0.0314 for CTA+MI (mean ± sample SD, *n* = 11). MI showed higher CKA than CTA-only and CTA+MI after correction (adjusted *p* = 0.02539 and 0.02148, respectively). The CTA-only versus CTA+MI difference did not remain significant (adjusted *p* = 0.20215). These values use the model-specific ACC reference extraction rules in Section 3.6.1; the cross-configuration differences are not interpreted as an isolated effect on native encoder representations.

The mean change in CKA after attention fusion was positive in all 11 participant-level summaries for both attention-based models: 0.2390 for CTA-only and 0.2393 for CTA+MI (adjusted *p* = 0.02148 for each comparison with zero). No statistically significant between-model difference was detected (adjusted *p* = 1.00000). The mean attention-output/query norm ratio was 0.4708 for CTA-only and 0.7557 for CTA+MI, with larger values for CTA+MI in all 11 participants (adjusted *p* = 0.02148). The attention branch therefore produced non-negligible output in the joint configuration, while the post-fusion representation showed greater structural similarity to the ACC reference.

#### 4.6.2. Class Geometry and Prediction Differences

Table 8 reports the class-scatter measurements. The mean within-to-between ratio was 0.5271 for CTA-only, 0.8048 for MI, and 0.5357 for CTA+MI within subjects; the corresponding values after adaptation were 0.7052, 0.8702, and 0.7090. CTA-only and CTA+MI each had a lower ratio than MI after correction (adjusted *p* = 0.00586 within subjects and 0.00781 after adaptation for both comparisons). The CTA-only versus CTA+MI ratio differences were not significant (adjusted *p* = 0.82617 in both settings). These global geometric rankings differed from the recognition rankings. Within-subject MI also had lower absolute within-class scatter, but its between-class scatter was lower, underscoring the need to consider both quantities.

**Figure 12:**
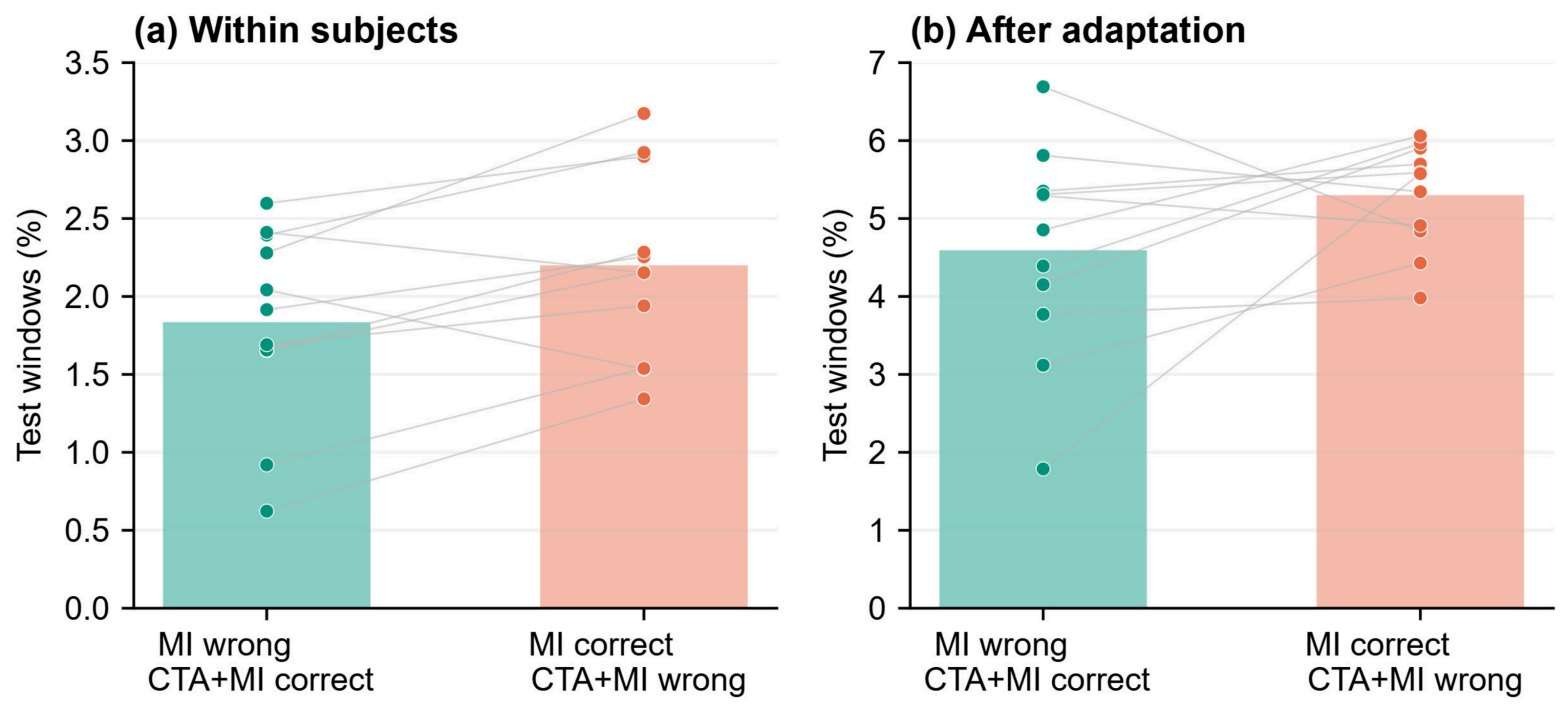
Prediction differences between MI and CTA+MI. Bars show group means and dots show each participant’s five-fold mean (*n* = 11). Both outcomes are percentages of all test windows; their difference equals the accuracy difference between the models.

Figure 12 shows the opposing prediction changes between MI and CTA+MI. Of all test windows within subjects, 1.83% were correctly classified by CTA+MI but misclassified by MI, whereas 2.20% were correctly classified by MI but misclassified by CTA+MI, yielding a net accuracy difference of −0.365 percentage points. Of all test windows after adaptation, 4.59% were correctly classified by CTA+MI but misclassified by MI, whereas 5.30% were correctly classified by MI but misclassified by CTA+MI, yielding a net accuracy difference of −0.706 points. CTA-only and MI also made different errors: after adaptation, 4.53% of all test windows were correctly classified only by CTA-only and 5.34% only by MI, while both models misclassified 9.85% of all test windows. Relative to CTA-only, CTA+MI had an average net difference of +1.161 points within subjects and +0.097 points after adaptation. Net accuracy differences are calculated before rounding the exclusive-correct percentages.

#### 4.6.3. Sensitivity to Attention Strength

Table 9 summarizes the fixed-weight interventions. Suppressing the attention update reduced accuracy in both models and both settings (adjusted *p* = 0.00781 for all four contrasts). The decreases were 29.19 and 25.54 percentage points within subjects for CTA-only and CTA+MI, respectively, and 41.29 and 43.35 points after adaptation. Halving the update reduced within-subject accuracy by 2.09 points for CTA-only and 0.59 points for CTA+MI (adjusted *p* = 0.00781 for both).

After adaptation, halving the update increased CTA-only accuracy from 84.81% to 85.40%, a gain of 0.587 points (adjusted *p* = 0.03711). CTA+MI increased from 84.91% to 85.31%, a gain of 0.405 points that was not significant after correction (adjusted *p* = 0.06738). Intervention gains are calculated from unrounded paired results. The effect of attenuating the attention update differed between within-subject recognition and recognition after adaptation.

### 4.7. Computational Cost and CPU Inference Latency

Table 10 quantifies the inference cost of the proposed MI model after removal of its training-only auxiliary modules. The classification network contained 121,034 parameters and required 7.582 MFLOPs per 300 ms window under the MAC-based counting convention in Section 3.4. Mean host-CPU forward latency was 0.4053 ms under the stated benchmark conditions.

## 5. Discussion

The matched comparison with Basic fusion provides the most direct evidence for the contribution of MI regularization. With the same dual-branch encoders and classification head, adding the MI objective increased mean accuracy by 6.00 percentage points before cross-subject adaptation and by 3.06 points after adaptation. A corresponding improvement of 2.52 points was observed within subjects. These differences remained significant under the reported Holm-adjusted configuration comparisons, supporting the empirical value of the additional dependence-learning objective in the evaluated framework.

Target adaptation contributes separately from MI regularization. The MI model improved from 37.02% to 85.61% accuracy after training on one complete target repetition of 40 gesture–posture trials. This 48.59-point change is an adaptation gain; the MI-specific matched gain under the same adaptation protocol is 3.06 points over Basic fusion. These results support multi-source learning followed by target-specific calibration, consistent with previous transfer and calibration studies [4,13,16,17,18,19,20]. MI’s accuracy SD decreased from 15.94 percentage points before adaptation to 3.88 afterward, consistent with reduced between-user performance dispersion after target-specific calibration. Inter-subject physiology, electrode placement, and movement execution are plausible contributors to the larger initial SD, but their separate effects were not quantified; this SD describes recognition variability, not sensor noise. The support repetition contains 40 labelled gesture–posture trials and 4720 overlapping windows, requiring 560 s (9 min 20 s) of scheduled recording, excluding device fitting, instructions, and operational delays. Separately, the network required approximately 0.4 ms per window on the tested host CPU (Table 10), quantifying its forward-execution cost. This timing excludes acquisition and preprocessing delays, and performance on resource-constrained hardware remains to be established.

The gesture-wise results show that adaptation improves mean recall and F1 across all ten gestures (Figure 5 and Table 3), while differences between gestures remain. Wrist extension, forearm supination, and wrist flexion were recognized most reliably after adaptation, whereas hand opening, thumbs-up, and the OK sign remained more difficult. The improvement therefore extends across the gesture set without eliminating class-dependent recognition difficulty. The posture-wise comparison likewise showed higher mean performance with MI than with Basic fusion across all four recorded arm postures (Table 5). Because these postures were represented in source training and target adaptation, the findings characterize performance across the evaluated posture conditions rather than generalization to unseen postures.

After adaptation, MI outperformed the evaluated local implementations of MSCNN-TL and the ACC-adapted Multistream CNN under the shared protocol. Before adaptation, its differences from these methods and sEMG-only recognition were not significant after correction. These comparisons concern the implementations and adaptation settings used here.

With both conditions fixed at 23,600 source windows, five-source pretraining exceeded single-source pretraining by 10.43 accuracy percentage points before adaptation and 1.09 points afterward. Equal sample count does not completely isolate subject diversity from source identity, signal quality, or other characteristics of the selected sources.

The original expectation of complementary recognition benefits from CTA and MI was not supported by the experimental findings. Adding CTA to MI did not increase mean recognition performance. MI exceeded CTA-only and CTA+MI significantly within subjects, but neither cross-subject difference remained significant after correction. Thus, under the evaluated conditions, the attention-free MI configuration provided the most favorable observed mean performance, while the broader effectiveness of attention-based fusion requires further investigation. The negative interaction contrasts describe a sub-additive performance pattern, not the significance of the direct MI-versus-CTA+MI difference or its cause.

Attention fusion increased CKA with the ACC reference, and CTA+MI retained a substantial attention output, but these representation changes did not correspond directly to higher recognition accuracy. CTA+MI also had a lower normalized within-to-between scatter ratio than MI in both settings despite its lower mean accuracy (Table 8 and Section 4.6.2). Fisher discriminant analysis evaluates separation along a chosen projection relative to within-class variation [32], whereas the ratio used here aggregates scatter across feature directions. Therefore, these geometric measurements should be interpreted as complementary representation analyses rather than direct explanations of recognition performance.

Paired predictions further show that similar mean performance can conceal different errors. Of all test windows after adaptation, 4.59% were correctly classified by CTA+MI but misclassified by MI, whereas 5.30% were correctly classified by MI but misclassified by CTA+MI. These opposing changes describe how the net accuracy difference arises across samples.

Fixed-weight interventions examine how trained attention models depend on their attention update. Suppressing that update substantially reduced accuracy in both configurations; this does not reproduce a separately trained attention-free model. Halving the update reduced within-subject accuracy but slightly increased accuracy after adaptation. For CTA+MI, the latter increase was 0.41 percentage points and was not significant after correction.

Partial overlap in the information used by CTA and MI remains a plausible explanation for limited incremental benefit. Multi-view learning theory considers redundant label-relevant information [33], while classification and MI objectives can interact through the shared encoders. Competing gradient directions [34] and the finite-sample properties of neural MI bounds [35,36] offer other possible influences. These diagnostics provide complementary observations of representation structure and prediction behavior, but they do not directly characterize the training mechanism responsible for the matched MI improvement.

All model comparisons and auxiliary analyses used the same cohort of 11 healthy, right-handed adults aged 20–27 years, including eight male and three female participants. Participant-level statistics avoid treating overlapping windows as independent observations but do not increase the number of independent participants. The MI-focused configuration comparisons and follow-up analyses remain exploratory and require validation in larger independent cohorts with broader age coverage and more balanced sex representation.

Several limitations should be considered when interpreting the findings. First, the current study focuses on offline steady-action recognition under controlled acquisition protocols. Extending the method to continuous interaction scenarios, including online gesture detection and transition-aware recognition, remains an important direction for future work. Second, although the MI-weight sensitivity analysis provides preliminary guidance for selecting the regularization strength, further studies are required to evaluate robustness under different training conditions and optimization settings. Third, validation across acquisition sessions, devices, preprocessing configurations, and larger user populations is needed to further assess the generalizability of the proposed method.

## 6. Conclusions

This study presented a dual-branch sEMG–ACC recognition method that combines supervised classification with cross-modal MI regularization. The MINE-based critic participates only in training, leaving an attention-free concatenation-and-classification architecture for inference. Relative to Basic fusion using the same recognition architecture without MI regularization, mean accuracy increased by 6.00 percentage points before cross-subject adaptation, 3.06 points after adaptation, and 2.52 points within subjects; all corresponding configuration comparisons remained significant after Holm correction. One-repetition adaptation achieved 85.61% accuracy and 85.57% Macro-F1, with performance above the evaluated local implementations of the transfer-learning baselines under the shared protocol.

Adding CTA provided no further mean recognition benefit, although the cross-subject difference between CTA+MI and MI was not significant after correction. The equal-budget experiment favored the tested multi-source compositions, and auxiliary analyses showed that recognition rankings cannot be inferred from global representation statistics alone. Together, these findings support MI regularization as an effective additional training objective in the evaluated cross-subject sEMG–ACC framework. Because the configuration ranking and follow-up analyses were obtained from the same dataset, independent validation across cohorts and acquisition conditions is still required to further confirm the generalizability of the observed benefits.

## Figures and Tables

**Table 1:** Status of the comparison implementations.

Method	Status/Implementation
Basic fusion	Matched ablation of this work; MI objective removed
CTA-only	Attention comparator implemented in this work
CTA+MI	Attention extension implemented in this work
MSCNN-TL	Local implementation adapted to the current sEMG input/output setting
Multistream CNN + FT	Local ACC-adapted implementation; not an exact reproduction of the reference IMU model

**Table 2:** Preliminary sensitivity analysis under strict LOSO before adaptation.

MI Weight λ	Accuracy (%)	Macro-F1 (%)
0	31.02	29.15
0.01	34.71	32.52
0.05	36.80	34.50
0.10	37.02	34.72
0.20	36.15	33.87

**Table 3:** Gesture-wise recall and F1 before and after one-repetition adaptation.

Gesture	Before Recall (%)	After Recall (%)	Before F1 (%)	After F1 (%)
G0 Hand open	18.7 ± 18.3	74.3 ± 8.5	17.9 ± 15.7	78.7 ± 6.9
G1 Fist	33.4 ± 23.7	86.1 ± 11.1	32.1 ± 18.6	86.7 ± 9.3
G2 Wrist flexion	52.9 ± 33.4	94.1 ± 4.5	49.5 ± 29.5	94.5 ± 4.2
G3 Wrist extension	60.7 ± 29.6	95.6 ± 2.6	59.5 ± 24.7	96.2 ± 2.0
G4 Forearm pronation	55.4 ± 28.1	92.8 ± 3.6	53.4 ± 28.3	93.8 ± 3.6
G5 Forearm supination	57.7 ± 31.3	94.4 ± 2.5	45.6 ± 27.3	93.8 ± 2.5
G6 Thumbs-up	15.1 ± 11.8	75.8 ± 12.7	15.9 ± 11.2	77.4 ± 11.5
G7 OK sign	20.8 ± 22.5	74.5 ± 13.7	19.3 ± 17.4	72.2 ± 10.9
G8 Index-finger extension	29.1 ± 22.7	82.1 ± 8.3	30.0 ± 20.4	78.1 ± 9.1
G9 V sign	27.1 ± 25.1	86.3 ± 8.4	24.6 ± 21.3	84.3 ± 7.9

Note. Values are mean ± sample SD across 11 target participants after the five support folds are averaged within each participant. Recall describes class-wise classification sensitivity.

**Table 4:** Cross-subject input-source and transfer-model comparisons under the same strict LOSO LAST-to-LAST protocol.

	Before Adaptation		After Adaptation	
Method	Accuracy (%)	Macro-F1 (%)	Accuracy (%)	Macro-F1 (%)
ACC-only	16.76 ± 5.56	14.63 ± 5.75	55.52 ± 4.10	54.88 ± 4.10
sEMG-only	30.00 ± 10.55	27.74 ± 10.30	74.04 ± 7.60	73.90 ± 7.69
Basic fusion	31.02 ± 13.56	29.15 ± 13.52	82.55 ± 4.36	82.52 ± 4.38
MSCNN-TL [14] (sEMG)	30.24 ± 13.36	29.19 ± 13.13	81.50 ± 6.33	81.42 ± 6.39
Multistream CNN + FT [15] (sEMG+ACC)	35.56 ± 16.21	33.40 ± 15.69	79.53 ± 5.91	79.36 ± 6.00
CTA+MI (sEMG+ACC)	35.32 ± 14.63	33.20 ± 14.45	84.91 ± 3.00	84.92 ± 2.98
MI (sEMG+ACC)	37.02 ± 15.94	34.72 ± 15.56	85.61 ± 3.88	85.57 ± 3.92

Note: MSCNN-TL uses sEMG and follows the multi-scale and source-domain adversarial framework of Fratti et al. [14], adapted to the local input/output dimensions. Multistream CNN + FT is a local ACC-adapted sEMG+ACC implementation of Li et al. [15], not an exact reproduction. Layer-freezing, batch-normalization, and adaptation settings are specified in Section 3.3. Values are mean ± sample SD across 11 target-subject five-fold summaries.

**Table 5:** Posture-wise performance of Basic fusion and MI under strict LOSO evaluation before and after one-repetition adaptation.

Evaluation Stage	Arm Posture	Basic Accuracy (%)	MI Accuracy (%)	Basic Macro-F1 (%)	MI Macro-F1 (%)
Before adaptation	Arm relaxed alongside the body	33.05 ± 13.34	37.92 ± 16.79	29.22 ± 13.80	33.60 ± 16.20
	Arm elevated forward to 45°	34.59 ± 17.11	40.13 ± 19.42	31.45 ± 16.24	36.44 ± 18.58
	Arm elevated forward to 90°	29.96 ± 17.13	38.71 ± 15.93	28.51 ± 16.42	35.75 ± 15.64
	Hand positioned in front of the eyes	26.42 ± 13.24	31.64 ± 16.22	22.56 ± 12.00	26.74 ± 14.93
After adaptation	Arm relaxed alongside the body	81.88 ± 5.07	85.90 ± 4.94	81.57 ± 5.15	85.63 ± 5.05
	Arm elevated forward to 45°	84.46 ± 3.97	86.52 ± 4.30	84.15 ± 4.13	86.10 ± 4.46
	Arm elevated forward to 90°	83.32 ± 5.18	85.95 ± 5.13	83.06 ± 5.17	85.65 ± 5.20
	Hand positioned in front of the eyes	80.56 ± 5.91	84.09 ± 5.88	80.03 ± 5.98	83.59 ± 6.11

Note. Accuracy and Macro-F1 are percentages reported as mean ± sample SD across 11 target participants, after averaging the five support-fold metrics within each participant. Both models were evaluated on the same posture-specific test windows. After adaptation refers to adaptation using one complete target repetition. These are descriptive posture-specific comparisons.

**Table 6:** Contribution of MI regularization relative to the matched Basic fusion ablation.

Evaluation Setting	Metric	Basic Fusion (%)	MI (%)	Gain (pp)	Holm-Adjusted *p*
Cross-subject before adaptation	Accuracy	31.02 ± 13.56 [21.91, 40.13]	37.02 ± 15.94 [26.31, 47.73]	+6.00	0.00293
	Macro-F1	29.15 ± 13.52 [20.07, 38.23]	34.72 ± 15.56 [24.27, 45.17]	+5.57	0.00879
Cross-subject after adaptation	Accuracy	82.55 ± 4.36 [79.62, 85.48]	85.61 ± 3.88 [83.00, 88.22]	+3.06	0.00293
	Macro-F1	82.52 ± 4.38 [79.58, 85.46]	85.57 ± 3.92 [82.94, 88.20]	+3.05	0.00293
Within subjects	Accuracy	91.26 ± 3.09 [89.18, 93.34]	93.78 ± 2.05 [92.40, 95.16]	+2.52	0.00586
	Macro-F1	91.17 ± 3.19 [89.03, 93.31]	93.74 ± 2.08 [92.34, 95.14]	+2.57	0.00586

Note. Accuracy and Macro-F1 are percentages, and gains are percentage-point differences between the displayed means. Basic-fusion and MI metrics are participant-level mean ± sample SD, followed in brackets by approximate 95% t confidence intervals for each group mean (mean ± t(0.975, 10) × SD/√11), calculated from the reported summaries. These intervals describe group-mean uncertainty, not paired-gain uncertainty. Gains are reported as unstandardized percentage-point differences. Adjusted *p* values use the configuration-comparison families separately for each metric: three MI-focused comparisons per cross-subject stage and six pairs within subjects (Section 3.3). The sampling unit is the participant’s five-fold mean (*n* = 11).

**Table 7:** Complete cross-subject configuration comparison before and after one-repetition adaptation using fixed final-epoch checkpoints.

	Before Adaptation		After Adaptation	
Model Variant	Accuracy (%)	Macro-F1 (%)	Accuracy (%)	Macro-F1 (%)
Basic fusion	31.02 ± 13.56	29.15 ± 13.52	82.55 ± 4.36	82.52 ± 4.38
CTA-only	35.23 ± 14.13	32.91 ± 13.87	84.81 ± 3.25	84.86 ± 3.25
CTA+MI	35.32 ± 14.63	33.20 ± 14.45	84.91 ± 3.00	84.92 ± 2.98
MI	37.02 ± 15.94	34.72 ± 15.56	85.61 ± 3.88	85.57 ± 3.92

**Table 8:** Class geometry of the classifier-input representations.

Setting	Configuration	*W*	*B*	*R*
Within-subject	CTA-only	0.325 ± 0.052	0.631 ± 0.061	0.527 ± 0.131
Within-subject	CTA+MI	0.328 ± 0.040	0.623 ± 0.045	0.536 ± 0.101
Within-subject	MI	0.231 ± 0.028	0.291 ± 0.023	0.805 ± 0.147
After adaptation	CTA-only	0.387 ± 0.039	0.556 ± 0.041	0.705 ± 0.122
After adaptation	CTA+MI	0.389 ± 0.034	0.555 ± 0.037	0.709 ± 0.108
After adaptation	MI	0.420 ± 0.060	0.496 ± 0.058	0.870 ± 0.212

Note. Values are mean ± sample SD of 11 participant-level five-fold means. Features were normalized separately to unit L2 norm; W and B are within-class and between-class scatter, and R = W/B. The measurements characterize the configurations described in Section 3.6.2.

**Table 9:** Accuracy (%) under fixed-weight attention interventions.

Setting	Configuration	α = 0	α = 0.5	α = 1	Holm-Adjusted *p* (α = 0.5 vs. 1)
Within-subject	CTA-only	63.06	90.16	92.25	0.00781
Within-subject	CTA+MI	67.88	92.82	93.41	0.00781
After adaptation	CTA-only	43.52	85.40	84.81	0.03711
After adaptation	CTA+MI	41.55	85.31	84.91	0.06738

Note. Values are means of 11 participant-level five-fold means. Alpha = 1 is the recomputed unaltered model; alpha = 0 and 0.5 suppress or halve the attention update. The final column gives Holm-adjusted p for alpha = 0.5 versus 1. All four alpha = 0 versus 1 comparisons had adjusted *p* = 0.00781; the eight contrasts form one correction family.

**Table 10:** Computational cost and host-CPU inference latency of the proposed MI model.

Model	Inference Parameters	FLOPs per 300 ms Window (M, 2 × MACs)	CPU Inference Latency (ms, Mean ± SD)
Proposed MI model	121,034	7.582336	0.4053 ± 0.0812

Note. The complete training model contains 195,275 parameters, including 74,241 parameters in the training-only ACC projection and MINE critic. Removing these auxiliary modules leaves 121,034 parameters for classification inference. FLOPs refer to twice the convolutional and linear-layer MAC count, excluding the additional scalar operations specified in Section 3.4; M denotes 10^6^. Latency is the mean ± sample SD of 5000 timed host-CPU forward passes under the conditions described in Section 3.4.

## Data Availability

The data presented in this study are available from the corresponding author upon reasonable request. The data are not publicly available due to privacy and ethical restrictions.

## Abbreviations

Abbreviation	Definition
sEMG	Surface electromyography
ACC	Acceleration
MI	Mutual information; MI regularization denotes the implemented dependence regularizer
MINE	Mutual Information Neural Estimation
CTA	Cross-temporal attention
LOSO	Leave-one-subject-out
IMU	Inertial measurement unit
CNN	Convolutional neural network
RNN	Recurrent neural network
LSTM	Long short-term memory
MSCNN-TL	Multi-scale convolutional neural network with transfer learning
FT	Fine-tuning
Conv1D	One-dimensional convolution
BN	Batch normalization
ReLU	Rectified linear unit
GAP	Global average pooling
CKA	Centered kernel alignment
SD	Sample standard deviation
CI	Confidence interval
LAST	Fixed final-epoch checkpoint
TP	True positive
FN	False negative
F1	Harmonic mean of precision and recall
pp	Percentage points
CPU	Central processing unit
FP32	32-bit floating-point precision
MAC	Multiply–accumulate operation
FLOPs	Floating-point operations
